# **Gut bacterial** ***O*****-demethylation modulates systemic exposure to oral etoposide**

**DOI:** 10.1080/19490976.2026.2628358

**Published:** 2026-02-13

**Authors:** Ashutosh Tripathi, Toe Ein Kyawt, Jongoh Shin, Kyoung-jae Won, Abigail T. Armstrong, Giokdjen Ilktach, Peter Sullivan, Holly A. Weilbaker, Yeonju Ko, Seongsoo Lee, Wooin Lee, Bruce R. Cooper, Byung-Kwan Cho, Jimmy Orjala, Hyunwoo Lee, Hyunyoung Jeong

**Affiliations:** a Department of Pharmaceutical Sciences and Center for Biomolecular Sciences, College of Pharmacy, University of Illinois at Chicago, Chicago, IL, USA; b Department of Industrial and Molecular Pharmaceutics, College of Pharmacy, Purdue University, West Lafayette, IN, USA; c Department of Biological Sciences, Chonnam National University, Gwangju, Republic of Korea; d College of Pharmacy and Research Institute of Pharmaceutical Sciences, Seoul National University, Seoul, Republic of Korea; e Metabolite Profiling Facility, Purdue University, West Lafayette, IN, USA; f Department of Comparative Pathobiology, College of Veterinary Medicine, Purdue University, West Lafayette, IN, USA; g Purdue Institute of Inflammation, Immunology and Infectious Disease, West Lafayette, IN, USA; h Department of Pharmacy Practice, College of Pharmacy, Purdue University, West Lafayette, IN, USA; i Purdue Institute for Cancer Research, West Lafayette, IN, USA

**Keywords:** Gut microbiota, *O*-demethylation, xenobiotic metabolism, etoposide, pharmacokinetics

## Abstract

Gut microbial *O*-demethylation has been reported for plant-derived dietary compounds containing *O*-methylated aromatic(s). However, the significance of gut microbial *O*-demethylation in drug metabolism and disposition remains unexplored. This study examined 64 clinically used oral drugs containing one or more methoxylated aromatics for gut microbial *O*-demethylation using high-resolution mass spectrometry (HRMS). For 35 of the tested drugs, including the anticancer agent etoposide, we detected metabolites corresponding to *O*-demethylation (i.e., a mass difference of −14 and its multiples) when individual drugs were incubated with mouse cecal contents. We confirmed that the *O*-demethylated metabolite (M1) of the model drug etoposide is etoposide catechol using HRMS and proton nuclear magnetic resonance spectroscopy. By testing an in-house collection of 56 gut bacteria individually, we identified seven previously unknown gut bacterial species that exhibit etoposide *O*-demethylating activity. Etoposide anticancer therapy has been associated with an increased risk of acute myeloid leukemia. We demonstrated that M1 is more genotoxic to myeloid cells when it is orally administered to mice, whereas M1 is less cytotoxic against MCF-7 and HeLa cancer cells than the parent etoposide, suggesting that the gut microbiota may contribute to the secondary genotoxicity of etoposide via *O*-demethylation. Comparative pharmacokinetic analysis of orally administered etoposide in control and antibiotic-treated mice showed that systemic exposure to etoposide increased 1.9-fold, while M1 exposure decreased 3.7-fold in antibiotic-treated mice, suggesting that gut microbial *O*-demethylation is a significant determinant of etoposide metabolism and disposition. Collectively, our study reveals the prevalence of gut bacteria with *O*-demethylation activity, illustrates the contribution of gut microbial *O*-demethylation to altering drug efficacy and toxicity with the model drug etoposide, and provides a knowledge basis for in-depth characterization of other drugs identified as being susceptible to gut microbial *O*-demethylation.

## Introduction

The mammalian gut microbiota metabolizes clinically used drugs.^1-3^ Resultant metabolites often exhibit unintended pharmacological activity and/or increased toxicity compared to their parent compounds. However, interindividual variations in the gut microbial composition and abundance challenge the outcome prediction of gut microbial drug metabolism. Identifying gut microbes and their genes involved in specific drug metabolism is critical to developing better microbiome-targeted and/or personalized therapies.

Recent studies have demonstrated the remarkable metabolizing capacity of gut bacteria, the predominant population of the mammalian gut microbiota, for hundreds of drug compounds with diverse chemical structures.^4^^,^^5^ In animal models and human studies, some of the gut bacterial drug metabolisms are shown to have clinical significance. For example, gut bacteria-mediated acetylation of 5-aminosalicylic acid lowers drug efficacy in patients with inflammatory bowel disease;^6^ decarboxylation of levodopa (followed by dehydroxylation) correlates with heterogeneous treatment outcomes in patients with Parkinson's disease;^7^ and inactivation of acarbose via hydrolytic degradation^8^ or phosphorylation^9^ results in reduced therapeutic efficacy in type-2 diabetic patients. These studies have demonstrated that individual variations in the abundance of gut bacteria that metabolize specific drugs impact systemic drug exposure, resulting in interindividual differences in therapeutic outcomes. On the other hand, the biochemical reactions responsible for gut bacterial drug metabolism are vastly diverse,^2-5^^,^^10^ and their contribution to drug metabolism and disposition remains to be determined in drug-specific manners.

*O*-demethylation (ODM) is one of the dealkylation reactions known to occur in the mammalian gut microbiota.^3^ ODM replaces a methyl group with a hydrogen atom in methoxylated aromatics (i.e., aryl methyl ethers), resulting in a net loss of a methylene group (i.e., CH_2_). Anaerobic environmental bacteria,^11-15^ termed acetogens, and methanogenic Archaea^16^ are relatively well-studied for their ODM activity, and most, if not all, characterized *O*-demethylases in these microorganisms are known to require the cobalamin cofactor (also known as corrinoid). In a few anaerobic gut bacteria, such as the acetogenic *Blautia producta* and *Eubacterium limosum*, ODM has also been shown to be mediated by corrinoid-dependent methyltransferase systems.^17^ Facultative anaerobic gut bacteria, such as *Enterococcus faecalis*, have been reported to have ODM activity, but the biochemical nature of the *O*-demethylase is unknown.^18^ The full breadth of ODM-capable gut bacteria and their genes encoding *O*-demethylases remains to be identified.

The gut bacterial ODM of plant-derived dietary or endogenous compounds, such as aryl methyl ethers (called *O*-methylated aromatics), produces bioactive metabolites. The gut bacterial ODM of the flavonoid isoxanthohumol generates the potent estrogenic metabolite 8-prenylnaringenin.^19^ ODM is an essential step in the concerted gut bacterial production of estrogenic lignan metabolites with chemopreventive activity against breast cancer.^20^^,^^21^ The gut bacterial ODM of the host-derived metabolite 3-methoxytyramine produces dopamine (a gut hormone that regulates gastrointestinal motility).^17^ While these studies suggest that the gut bacterial ODM of naturally occurring compounds is likely prevalent in individuals, the impact of gut bacterial ODM on drug metabolism and disposition remains largely unknown.

Given the significant similarity in chemical structures between natural compounds and drugs, we hypothesize that drug compounds containing *O*-methylated aromatic(s) are *O*-demethylated by the gut microbiota. Here, we report that drugs with diverse chemical structures in different therapeutic classes undergo gut microbial ODM. In particular, we show that gut microbial ODM is a previously unrecognized elimination route for the anticancer agent etoposide in mice, significantly contributing to systemic exposure to the parent drug and its *O*-demethylated metabolite. Moreover, we identified previously unknown gut bacteria in which *O*-demethylate etoposide. This study demonstrated that gut bacterial ODM is a major determining factor for the metabolism and disposition of the prescription drug etoposide, and it provides a list of candidate drugs for an in-depth investigation of gut microbial ODM.

## Results

### Identification of drugs potentially metabolized via gut microbial *O*-demethylation

**Figure 1.**
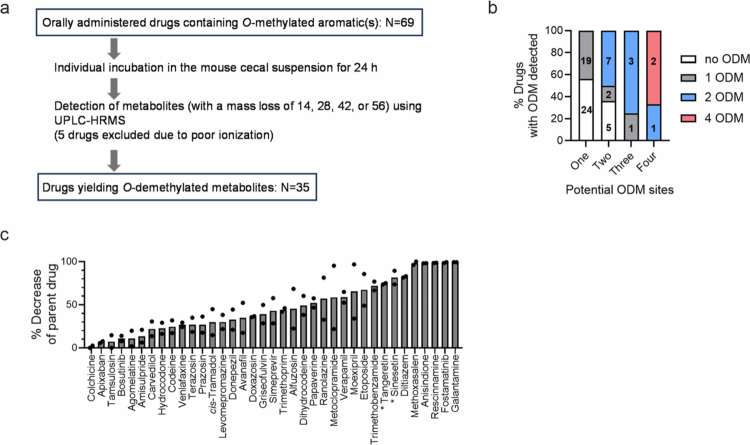
Identification of drugs *O*-demethylated by the gut microbiota. (a) Schematic for the screening of drugs subjected to gut microbial ODM (*n* = 3). (b) Distribution of 35 ODM-positive drugs among 65 drugs analyzed for gut microbial ODM. In each column, the number of drugs in each category is shown. (c) Overall gut microbial metabolism of 35 ODM-positive drugs. Respective drugs were individually incubated in the mouse cecal suspension from two different mice for 24 h, followed by measurement of the parent drug disappearance using HPLC–UV. The average values of the biological duplicates (*n* = 2) are shown, and marked with a star (*) represent two positive control compounds.

To identify drug substrates for gut microbial ODM, we first retrieved FDA-approved drugs containing *O*-methylated aromatic(s) from the DrugBank database (www.cheminfo.org).^22^ Of the 105 drugs retrieved, we initially focused on 69 orally administered drugs (Figure 1a). Two botanical compounds (sinensetin and tangeretin), which were previously shown to undergo gut bacterial ODM,^23^ were included as positive controls; each compound has five potential ODM sites. The effects of the 69 drugs and two control compounds on the gut microbial ODM were tested by individually incubating them (50 µM) in the mouse cecal suspension for 24 h, followed by ultra-performance liquid chromatography–high resolution mass spectrometry (UPLC–HRMS) analysis. Five drugs (acitretin, mestranol, methicillin, nabumetone, and naproxen) were excluded from the analysis due to MS incompatibilities (e.g., poor ionization of parent drugs), and the remaining 66 drugs (64 drugs plus two positive controls) were analyzed for the appearance of *m/z* peak(s) with a mass difference of −14 (i.e., demethylation; loss of -CH_2_) and/or multiples within a mass error of <10 ppm. Tangeretin produced three *m/z* peaks corresponding to metabolites with single, double, and triple ODM (i.e., mass difference of −14, −28, and −42, respectively), and sinensetin yielded a single *m/z* peak corresponding to a mass difference of −28 (i.e., double ODM), validating our methodology for the detection of gut microbial ODM. Approximately 55% of the tested drugs (35/64) yielded *m/z* peak(s) corresponding to *O*-demethylated metabolites in at least two of the three independent experiments (Table S1 for UPLC–HRMS data).

The 64 drugs tested for gut microbial ODM included 43 drugs with one potential ODM site, 14 with two ODMs, four with three ODMs, and three with four ODMs (see Figure S1 for chemical structures); 35 drugs were ODM-positive under our test conditions (Figure 1b and Table 1). The majority (22) of the 35 ODM-positive drugs were found to be singly *O*-demethylated, including 19 with one ODM site, two with two ODMs, and one with three ODMs. We did not observe any notable differences in chemical features between 19 ODM-positive drugs with one ODM site and 24 ODM-negative drugs with one ODM site. Among the 14 drugs with two ODM sites, five were ODM-negative, and nine ODM-positive drugs included two drugs yielding a single ODM and seven drugs with both single and double ODM detected. Notably, six of the latter seven ODM-positive drugs contained two methoxy groups *ortho*-positioned to each other on the same aromatic ring (alfuzosin, donepezil, doxazosin, moexipril, prazosin, and terazosin), and one drug (etoposide) had two methoxy groups *meta*-positioned to each other with a hydroxy group in the middle (see below in this paragraph). In contrast, four of the five ODM-negative drugs with two ODM sites contained two methoxy groups either distantly (i.e., *meta* or *para* position to each other) on the same aromatic ring or each on a different aromatic ring. An exception to this observation was tetrabenazine, an ODM-negative drug with *ortho*-positioned two ODM sites. While four drugs, which harbor three methoxy groups on the same aromatic ring, are ODM-positive, their ODM patterns were different: two drugs (colchicine and trimethobenzamide) yielded both single and double ODM, one drug (trimethoprim) yields single and triple ODM, and one drug (fostamatinib) yields only single ODM. Fostamatinib is a prodrug whose dephosphorylation produces an active form (tamatinib) of the drug,^24^ and we detected only an *m/z* peak corresponding to a single ODM of tamatinib but not fostamatinib, suggesting that the gut microbiota also efficiently mediates dephosphorylation. Three drugs with four ODM sites were all ODM positive, with a notable difference between one drug (rescinnamine) and two drugs (papaverine and verapamil). Rescinnamine contains three methoxy groups on the same aromatic ring and one methoxy group on another aromatic ring, and we observed two *m/z* peaks for single and double ODM. In contrast, papaverine and verapamil contain two aromatic rings, each carrying *ortho*-positioned two methoxy groups, and they produced four *m/z* peaks for single, double, triple, and tetra ODM, suggesting that creating a hydroxy group by ODM at one position may facilitate the ODM of another at the *ortho* position. In line with this notion were seven ODM-positive drugs: six drugs containing *ortho*-positioned two ODM sites (alfuzosin, donepezil, doxazosin, moexipril, prazosin, and terazosin) and etoposide harboring *meta*-positioned two ODM sites with a hydroxy group in the middle.

**Table 1.** Drugs detected to be *O*-demethylated by the gut microbiota.

Drug names	No. of potential ODM sites	No. of ODM detected
Agomelatine	1	One
Amisulpride^a^	1	
Anisindione	1	
Apixaban	1	
Avanafil	1	
Bosutinib	2	
Carvedilol^a,b,c^	1	
Codeine	1	
Dihydrocodeine	1	
Diltiazem^a^^,^^b^	1	
Fostamatinib	3	
Galantamine^a^	1	
Griseofulvin^a^	2	
Hydrocodone	1	
Levomepromazine	1	
Methoxsalen^a^^,^^b^	1	
Metoclopramide^a^	1	
Ranolazine^a,^^b^	1	
Simeprevir	1	
Tamsulosin^a^^,^^b^	1	
*cis*-Tramadol^b^	1	
Venlafaxine^a^^,^^b^	1	
Alfuzosin^a^^,^^b^	2	Two
Colchicine^a^^,^^b^	3	
Donepezil^b^	2	
Doxazosin^a,b,c^	2	
Etoposide^b^	2	
Moexipril	2	
Prazosin^a^^,^^b^	2	
Rescinnamine	4	
Terazosin^a^^,^^b^	2	
Trimethobenzamide^a,b,c^	3	
Trimethoprim^b^	3	
Papaverine^a^	4	Four
Verapamil^a^^,^^b^	4	

a,b,c Other studies have also tested for gut bacterial metabolism (see Discussion):

a Zimmermann et al. ^5^;

b Javdan et al. ^4^; and

c Wu et al. ^25^.

To gain a general understanding of the extent of gut microbial metabolism of 35 ODM-positive drugs, each compound (100 µM) was incubated in the mouse cecal suspension, and its disappearance was determined using high-performance liquid chromatography–ultraviolet spectroscopy (HPLC–UV). We observed a wide range of gut microbial metabolism from minimal (colchicine) to almost complete (anisindione, fostamatinib, galantamine, methoxsalen, and rescinnamine) disappearance of a parent compound (Figure 1c). Together, these results provide a list of candidate drug compounds for in-depth investigation of gut microbial ODM and its contribution to overall gut microbial drug metabolism, pharmacokinetics, and pharmacodynamics.

### Gut bacteria *O*-demethylate etoposide

**Figure 2.**
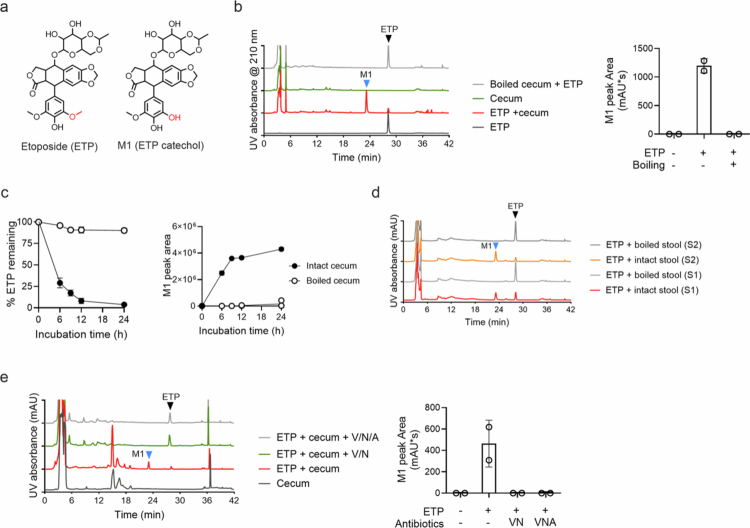
Gut bacterial *O*-demethylation of etoposide. (a) Structures of etoposide and its *O*-demethylated metabolite (M1: etoposide catechol). (b) Etoposide (ETP, 100 μg/mL) was incubated in a mouse cecal suspension (50 mg/mL each) under anaerobic conditions for 24 h, followed by HPLC‒UV analysis. The M1 peak areas from two independent experiments are shown (*n* = 2). (c) Time profiles of etoposide disappearance and M1 appearance when etoposide was incubated with intact or boiled mouse cecal suspension. The means ± SD are shown. (*n* = 3) (d) Intact or boiled human stool samples from two subjects (S1 and S2) were incubated with etoposide (ETP, 100 μg/mL) under anaerobic conditions for 24 h, followed by HPLC–UV analysis. (e) Mouse cecal suspension (50 mg/ml) was pretreated with a cocktail of antibiotics (V, vancomycin; N, neomycin; A, ampicillin; 0.1 g/L each) for 3 h, washed, and resuspended in pre-reduced PBS. An intact or antibiotic-treated human stool sample was incubated with etoposide (ETP, 25 μg/mL) under anaerobic conditions for 4 h, and the reaction mixtures were analyzed using HPLC–UV. The arrowheads indicate etoposide (ETP; black) and M1 (blue).

Among the 35 ODM-positive drugs identified, we chose to investigate the anticancer agent etoposide^26^ in-depth since its *O*-demethylated metabolite “etoposide catechol” is commercially available (Figure 2a). Notably, the oral bioavailability of etoposide is known to exhibit high inter-individual variability, ranging from 25% to 97%.^27^^,^^28^ The underlying causes of this variability remain unknown, possibly suggesting the involvement of the gut microbiota in etoposide metabolism.

In the screen for gut microbially *O*-demethylated drugs above, the qualitative UPLC‒HRMS analysis detected *m/z* peaks for two possible metabolites arising from the single and double ODM of etoposide when incubated in the mouse cecal suspension. We further characterized gut microbial etoposide metabolism quantitatively. When etoposide was incubated with the mouse cecal suspension for 24 h, followed by analysis of the reaction mixture using HPLC–UV, a new peak named “M1” was observed (Figure 2b). The M1 peak was not observed when etoposide was incubated with a pre-boiled mouse cecal suspension. M1 production increased over time and coincided with a decrease in the parent etoposide (75% decrease at 6 h and 90% at 12 h of incubation) (Figure 2c). We also examined etoposide metabolism by the human gut microbiota. When etoposide was incubated with fresh stool samples from two human subjects for 24 h, we observed M1 production but not with boiled stool samples (Figure 2d). These results suggested that M1 is a primary metabolite of etoposide produced by the mouse and human gut microbiotas.

To determine whether gut bacteria mediate etoposide metabolism to M1, the mouse cecal suspension was pre-treated with an antibiotic cocktail (vancomycin and neomycin; or vancomycin, neomycin, and ampicillin at 0.1 g/L each) for 3 h, washed with pre-reduced PBS, and incubated anaerobically with etoposide for 4 h, and the reaction mixture was analyzed using HPLC–UV. The M1 peak was not observed in the antibiotic-treated samples (Figure 2e), suggesting that gut bacteria are required for M1 production.

To elucidate the M1 chemical structure, M1 was mass-produced by incubating etoposide in a human stool suspension, which was subsequently isolated through solid-phase extraction, purified using HPLC, and analyzed by HRMS and nuclear magnetic resonance (NMR) spectroscopic methods. The *m/z* values for etoposide and purified M1 were [M+NH_4_]^+^ 606.2193 and 592.2036, respectively (Figure S2A), which were consistent with the chemical formulas for etoposide (C_29_H_36_NO_13_ with a calculated mass of 606.2181 Da) and for M1 (C_28_H_34_NO_13_ with a calculated mass of 592.2025 Da). The expected chemical formula suggested that M1 is a demethylated product (i.e., removal of -CH_2_) of etoposide, and the fragmentation pattern of M1 indicated that demethylation occurred in the pendant ring (Figure S2A). Proton-NMR spectra further confirmed that the purified M1 was etoposide catechol (Figure S2B). Together with the requirement of gut bacteria for etoposide metabolism by the gut microbiota, this result confirmed that the gut bacterial metabolism of etoposide primarily produces an *O*-demethylated metabolite.

We also determined whether etoposide analogs (podophyllotoxin and teniposide) are associated with gut microbial ODM. The incubation of the respective etoposide analogs with the mouse cecal suspension resulted in the production of metabolites with mass differences of −14 and/or −28 (Figure S3A,B), indicating the gut microbial ODM of the etoposide analogs. These results suggest that the gut microbiota converts etoposide and its structural analogs to their catechol metabolites via ODM.

### Identification of etoposide *O*-demethylating gut bacteria

**Figure 3.**
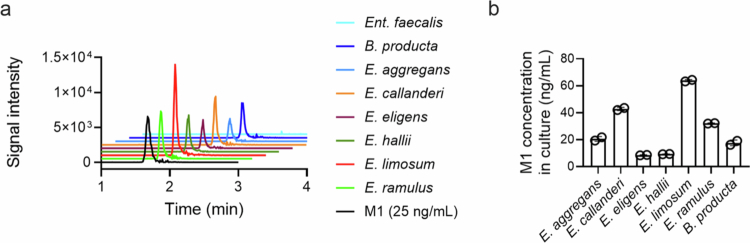
Identification of the gut bacteria *O*-demethylating etoposide. The cells of the respective bacterial cultures were harvested and resuspended to an OD_600_ of 2 and incubated with etoposide (10 μg/mL) anaerobically for 24 h, followed by LC‒MS/MS analysis. (a) Representative LC‒MS/MS chromatograms. (b) M1 concentrations in the culture supernatants (*n* = 2).

Several gut bacteria, such as *B. producta*, *Ent. faecalis*, *E. callanderi*, and *E. limosum*, are known to mediate ODM of botanical (biochanin A, formononetin, isoxanthohumol, and secoisolariciresinol) and endogenous (3-methoxytyramine) compounds.^17^^,^^18^^,^^20^^,^^29-31^ We tested whether these four previously known gut bacteria mediate etoposide ODM. The cells were harvested from individual cultures of four bacteria grown in a brain heart infusion medium supplemented with cysteine (BHIc), washed with pre-reduced PBS, and resuspended at an optical density of 2 at 600 nm (OD_600_) in a modified M9 medium. After the cell resuspension was incubated with etoposide (10 µg/mL) for 24 h, M1 production was determined using HPLC–MS/MS. Three gut bacteria (*B. producta*, *E. callanderi*, and *E. limosum*) but not *Ent. faecalis*, produced M1 (Figure 3), while the respective control cultures without added etoposide did not (data not shown). Notably, the three etoposide *O*-demethylating bacteria displayed a conversion of 0.2%–0.6% of the parent etoposide to M1 under our test conditions. This finding was in sharp contrast to the almost complete ODM of etoposide in the mouse cecal suspension (Figure 2c), possibly indicating the presence of as-yet-unknown gut bacteria that *O*-demethylate etoposide. To examine this possibility, we tested an in-house collection of 52 gut bacteria for etoposide ODM (Table S2). These 52 gut bacteria were previously unknown for ODM activity, and we identified four gut bacteria (*E*. *aggregans*, *E. eligens*, *E. hallii*, and *E. ramulus*) could *O*-demethylate etoposide (Figure 3). These results suggest that structurally different botanical and drug compounds containing *O*-methylated aromatics can be metabolized by the same gut bacteria and reveal previously unknown *O*-demethylating gut bacteria.

Among the seven gut bacteria identified to *O*-demethylate etoposide above, *E. limosum* was the only bacterium for which genetic manipulation tools were developed,^32^ and it is closely related to another etoposide *O*-demethylating bacterium *E. callanderi,* whose genome was previously proposed to harbor six putative ODM systems.^29^^,^^33^ Each ODM system consists of four proteins, including methyltransferase-I (MT-I), corrinoid protein, methyltransferase-II, and activating enzyme, and MT-I catalyzes the demethylation of an ODM-susceptible compound and defines substrate specificity.^34^ Each of the five ODM systems in *E. callanderi* contains an MT-I, and one has two MT-Is. To identify an MT-I that *O*-demethylates etoposide in *E. limosum*, we performed a tBLASTn search with seven respective *E. callanderi* MT-I proteins as bait in the genome of *E. limosum*. As a result, four *E. limosum* MT-I homologs that exhibit 97.8%–98.5% identities in the overall amino acid sequences were retrieved, and many homologs with lower percent identities ranged from approximately 30%–53% (Table S3). We focused on the four MT-I homologs with high identities and attempted to construct the corresponding MT-I mutants in *E. limosum* using CRISPR-Cas9-based mutagenesis.^32^ We were able to obtain three mutants (Figure S4A), each disrupted for an MT-I gene, but were unable to obtain a fourth mutation despite multiple attempts. The three MT-I mutants exhibited wild-type growth in a test medium (data not shown); however, their etoposide *O*-demethylase activity was comparable to that of the wild-type strain (Figure S4B). While we cannot rule out the possibility that the MT-1 gene whose disruption could not be obtained might *O*-demethylate etoposide, this result suggested that none of the three deleted MT-Is are responsible for etoposide ODM, that multiple MT-Is have redundant functions and compensate for the loss of one, and/or that MT-I protein(s) with lower homology mediate etoposide ODM in *E. limosum*.

### M1 (etoposide catechol) is less cytotoxic against cancer cells but more genotoxic to bone marrow cells

**Figure 4.**
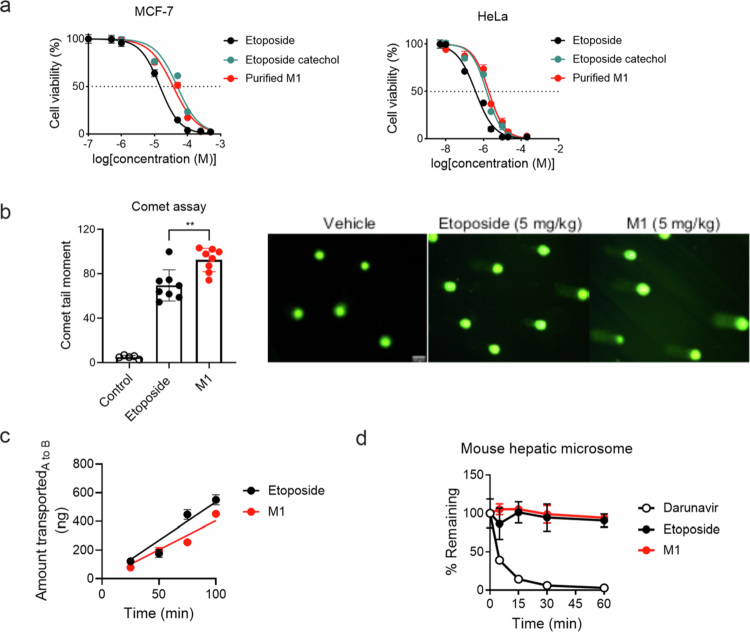
M1 is less cytotoxic but more genotoxic than the parent etoposide. (a) Etoposide and M1 cytotoxicity were determined against MCF-7 (human breast cancer cells) and HeLa (human cervical cancer cells) using sulforhodamine B assay (*n* = 3). (b) Comet assays were performed to determine DNA damage. A group of mice (*n* = 5–8/group) was treated with vehicle, etoposide, or M1 (5 mg/kg, i.v.), and an alkaline comet assay was performed. To quantify DNA damage, the comet tail moment was measured using TriTek CometScore software. Representative images of comet tails are shown. Scale bar, 50 μm. **, *p* < 0.01. (c) Apical to basolateral transport rates of etoposide and M1 across the Caco-2 monolayer were measured (*n* = 2). (d) Hepatic metabolism of etoposide and M1 (1  μM) was determined using mouse hepatic microsomes (0.5 mg/mL) in duplicates. Darunavir is a drug known to be rapidly metabolized by hepatic drug-metabolizing enzymes and is used as a control. Drug concentrations were measured using HPLC–MS/MS (*n* = 2). All the data are represented as the mean ± SD.

To evaluate the pharmacological activities of M1 (i.e., etoposide catechol) and etoposide, we determined their cytotoxicity against HeLa and MCF-7 cells using sulforhodamine B (SRB) colorimetric assay. The half maximal inhibitory concentrations (IC_50_) of etoposide, etoposide catechol (purchased from Sigma), and M1 (isolated for structural determination) were 14.7, 48.9, and 36.6 µM for MCF7 cells and 0.4, 1.5, and 1.9 µM for HeLa cells (Figure 4a). Notably, the IC_50_ values of M1 were comparable to those of commercial etoposide catechol. These results demonstrate that M1 (i.e., etoposide catechol) is 3~5-fold less cytotoxic than the parent etoposide against cancer cells.

Etoposide, as an anticancer agent, inhibits topoisomerase II, blocking re-ligation after DNA cleavage.^35^ On the other hand, etoposide treatment is associated with an increased risk of acute myeloid leukemia,^26^ and etoposide catechol has been shown to be more potent than the parent etoposide in triggering DNA strand breaks in biochemical assays *in vitro*.^36^ However, the biological consequences of the activity of etoposide catechol *in vivo* are unknown. To compare the genotoxicity of etoposide catechol and etoposide *in vivo*, a single-cell gel electrophoresis comet assay was performed after the administration of etoposide or etoposide catechol to the mice. The mice were treated with etoposide or etoposide catechol (5 mg/kg, intravenously), and bone marrow cells were collected from the femurs after one hour and subjected to the comet assay. A significant increase in the mean comet tail moment was observed in mice treated with etoposide catechol compared to those treated with etoposide (Figure 4b), indicating that etoposide catechol (i.e., M1) causes more DNA strand breaks than etoposide *in vivo*.

### Etoposide and M1 exhibit comparable absorbability and hepatic metabolism

To estimate the absorbability of M1 produced by gut bacteria in the intestine, we performed a transport study using human Caco-2 cells grown on a transwell. After the Caco-2 cell monolayer formed, M1 or etoposide (10 µM) was added to the apical chamber, and the appearance rate of M1 macrophages in the basolateral chamber was measured. The estimated apparent permeability values (*P*_app_) of etoposide and M1, 17.2 ± 1.2 × 10^−6^ cm/s and 13.1 ± 0.2 × 10^−6^ cm/s, were of the same order of magnitude (Figure 4c), suggesting that M1 likely exhibits similar gut absorption as etoposide.

All compounds absorbed from the intestine pass through the liver, the major organ for drug metabolism, before reaching the systemic circulation. To estimate the rate of hepatic metabolism, M1 was incubated with hepatic microsomes. Darunavir, a compound readily metabolized by cytochrome P450s, was included as a control, and etoposide was included for comparison. The darunavir concentration decreased rapidly over time, as expected, but etoposide elimination was minimal in hepatic microsomes, which is consistent with a previous study reporting very low hepatic extraction of etoposide (Figure 4d).^26^ The M1 disappearance rate was also slow, similar to that of etoposide (Figure 4d), suggesting that minimal hepatic oxidative metabolism of M1.

### The gut microbiota is a major determinant of systemic exposure to orally administered etoposide

**Figure 5.**
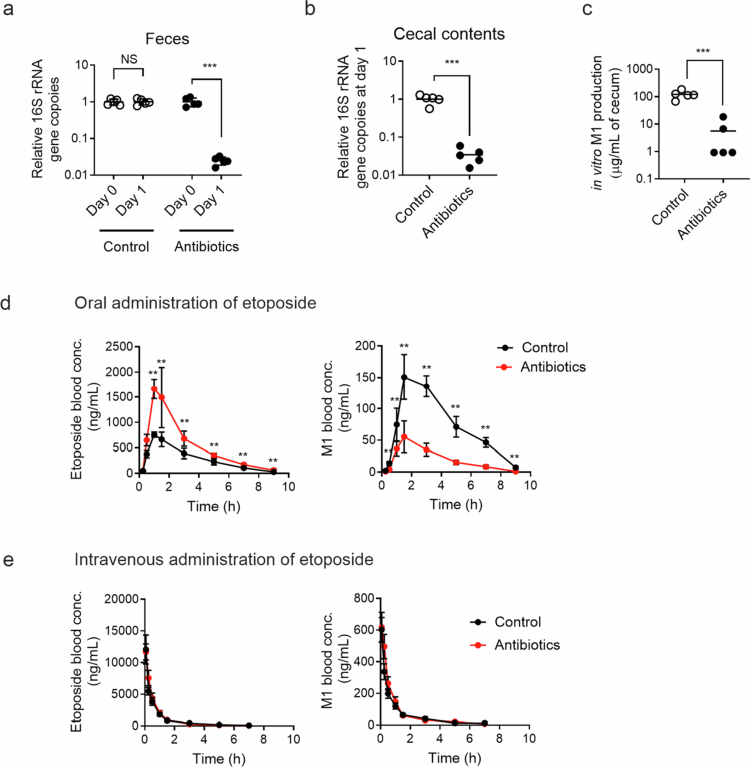
The gut microbiota governs the systemic exposure of orally administered etoposide. C57BL6/J mice were pretreated with antibiotic- containing water (0.5 g/L vancomycin and 0.1 g/L polymyxin B) or regular water for 24 h (a and b; *n* = 5). The bacterial abundance in the fecal pellets (a) and cecal contents (b) was determined by measuring the relative number of copies of the 16S rRNA gene before (day 0) and after (day 1) antibiotic administration. (c) Etoposide (100  μg/mL) was incubated in the cecal contents of antibiotic-pretreated or control mice, and after 3 h, the M1 concentration was measured using HPLC–MS/MS (*n* = 5). M1 production was normalized by the amount of cecal contents. (d and e) After 24 h of antibiotic treatment, the mice were fasted for 3 h, followed by oral (20 mg/kg; d) or intravenous (10 mg/kg; e) administration of etoposide (*n* = 9–10/group). Blood was collected at multiple time points for measuring etoposide and M1 using HPLC–MS/MS. **; *p* < 0.001 *vs*. control. All the data are represented as the mean ± SD.

Whether gut microbial drug metabolism contributes to systemic exposure to an orally administered drug can be determined by comparing pharmacokinetic profiles between antibiotic-treated and control-conventional mice. However, antibiotic treatment for as short as 5 d is known to affect the hepatic expression of genes involved in etoposide elimination.^37^ Therefore, we first established an antibiotic treatment protocol for our study. Treatment of mice with non-absorbable oral antibiotics (0.5  g/L vancomycin and 0.1 g/L polymyxin B in drinking water) for 24 h led to a >95% reduction in the content of the bacterial 16S rRNA gene in both mouse cecum and fecal samples (Figure 5a,b), with no changes in the mRNA levels of etoposide-metabolizing enzymes (*Cyp3a11* and *Ugt1a1*)^38^ and etoposide-transporting efflux pump transporters (*Abcb1a, Abcc2*, and *Abcc3*)^39^ in the liver and small intestine (Figure S5A). When the caecal suspension from antibiotic-treated mice was incubated with etoposide (100  µg/mL), M1 production was significantly decreased (Figure 5c), suggesting that one-day antibiotic treatment effectively eliminates etoposide *O*-demethylating gut bacteria in these mice. Antibiotic treatment can compromise the integrity of the intestinal tight junction barrier,^40^^,^^41^ which may affect the intestinal absorption of a drug. To determine whether 24-h antibiotic treatment affects gut integrity (and potentially increases the intestinal absorption of etoposide), we examined the integrity of the intestinal tight junction barrier in antibiotic-treated mice by orally administering FITC-dextran (4 kDa) and determining the rate of its appearance in the systemic circulation.^42^ FITC-dextran concentrations in mouse plasma were similar between the control and antibiotic-treated mice (Figure S5B). Additionally, the mRNA expression levels of tight junction components (*Tjp-1*, *Ocln*, and *Muc-2*) were similar between the two groups (Figure S3A), suggesting that 24-h antibiotic treatment does not affect the intestinal absorption of etoposide. These results suggest that one-day antibiotic treatment minimally impacts the host absorption and elimination of etoposide while significantly reducing M1 production by the gut microbiota.

With the one-day antibiotic-treatment protocol established, we investigated the contribution of gut bacterial ODM to overall etoposide disposition by comparing pharmacokinetic profiles between antibiotic-treated and control mice. Etoposide (20 mg/kg) was administered via oral gavage to the antibiotic-pretreated mice, and blood samples were collected over time. The maximum blood concentration (C_max_) and the area under the curve (AUC_0–∞_) of etoposide were 2.3- and 1.9-fold higher in antibiotic-treated mice compared to control mice, respectively (Figure 5d and Table 2). On the other hand, the C_max_ and AUC_0–∞_ of M1 were 2.6- and 3.7-fold lower, respectively, in the antibiotic-treated mice. We noted that within 2 h after oral administration of etoposide, M1 reached the maximum blood concentration, approximating the gastric emptying time of the mice,^43^ suggesting the presence of etoposide ODM by small intestinal gut bacteria. To determine whether the conversion of etoposide to M1 occurs in the small intestine (in addition to the large intestine), the samples were incubated with etoposide (100 µg/mL) to wash out the resected mouse jejunum and ileum for 6 h. The mouse cecal suspension was included for comparison, and both samples (small intestine and cecum) were normalized to OD_600_ of 2. The conversion of etoposide to M1 by the small intestinal suspension (~10%) was significant, albeit lower than that by the cecal suspension (~34%) (Figure S5C), indicating that mouse small intestinal bacteria also catalyze the ODM of etoposide. To further rule out the effects of antibiotic treatment on host elimination of etoposide (or M1), etoposide (10 mg/kg) was administered intravenously via the tail vein to antibiotic-pretreated mice, followed by blood sampling at multiple time points. The pharmacokinetic parameters, including the terminal half-life (t_1/2_) and AUC_0-∞_, were similar between antibiotic-treated and control mice receiving etoposide intravenously (Figure 5e and Table 2). This result is consistent with data showing minimal effects of antibiotics on the intestinal and hepatic expression of genes involved in etoposide elimination (Figure S5A) and with a previous study reporting insignificant intestinal and biliary excretion of host-derived etoposide metabolites (e.g., etoposide glucuronide).^39^ Together, these results suggest that gut bacterial metabolism of etoposide to M1 via ODM is a significant determinant of etoposide (and M1) systemic exposure upon oral administration of etoposide.

**Table 2.** Pharmacokinetic parameters of etoposide and M1 after intravenous or oral administration of etoposide in mice (*n* = 9–10/group).

	Control	Antibiotics treatment
**Intravenous** (10 mg/kg)		
**Etoposide**		
AUC_0–∞_ (ng/mL·h)	8020 ± 407	8534 ± 417
C_0_ (ng/mL)	18187 ± 5307	14755 ± 3201
Terminal t_1/2_ (h)	1.64 ± 0.28	2.15 ± 0.80
**M1**		
AUC_0–∞_ (ng/mL·h)	512 ± 37	576 ± 39
C_0_ (ng/mL)	816 ± 169	697 ± 145
Terminal t_1/2_ (h)	1.7 ± 1.0	2.1 ± 0.7
**Fractional AUC** (M1/etoposide)	0.064 ± 0.004	0.068 ± 0.006
**Oral** (20 mg/kg)		
**Etoposide**		
AUC_0–∞_ (ng/mL·h)	2653 ± 243	5053 ± 734****
C_max_ (ng/mL)	777 ± 76	1816 ± 371****
T_max_ (h)	1.1 ± 0.2	1.2 ± 0.3
Terminal t_1/2_ (h)	1.4 ± 0.3	1.2 ± 0.3
Bioavailability (%)	16.5	29.6
**M1**		
AUC_0–∞_ (ng/mL·h)	689 ± 51	188 ± 20****
C_max_ (ng/mL)	158 ± 31	61 ± 18****
T_max_ (h)	2.0 ± 0.8	1.7 ± 0.8
Terminal t_1/2_ (h)	1.4 ± 0.3	1.2 ± 0.3
**Fractional AUC** (M1/etoposide)	0.261 ± 0.023	0.038 ± 0.07

• Data analyzed by non-compartmental analysis (NCA) using WinNonlin software (Certara, version 5.0.1.). • All the data are represented as the mean ± SD.

**** *p* < 0.0001.

## Discussion

The gut microbial ODM of clinically used drugs and its clinical significance in drug therapy has largely been unknown. In this study, we found that more than half of the tested drugs (35/64) containing one or more *O*-methylated aromatic groups are metabolized by the gut microbiota, producing metabolite(s) with a mass difference of −14 and/or its multiples corresponding to demethylation. This qualitative result suggests a previously unappreciated prevalence of gut microbial ODM associated with drugs, and it provides candidate drugs to study the overall impact of gut microbial ODM on the systemic drug exposure.

As illustrated in this study, our qualitative screening result requires quantitative verification that determines the contribution of gut microbial ODM to overall gut microbial drug metabolism. When the disappearance of parent compounds during incubation in the mouse cecal suspension was examined, some ODM-positive drugs showed minimal gut microbial metabolism. This observation suggests that while the ODM of these drugs can be detected by highly sensitive methods such as HRMS, its contribution to the overall gut microbial drug metabolism may be insignificant. On the other hand, our data showing, for example, less than 50% in the overall gut microbial drug metabolism over 24 h of incubation under our test conditions, needs cautionary interpretation. While this result may suggest a minor contribution to the pharmacokinetics of the parent drug (i.e., systemic exposure) in physiological settings, such low conversion *in vitro* does not necessarily imply physiological irrelevance *in vivo*, particularly when the gut bacterial metabolism of a drug does not alter systemic exposure to the parent drug but produces a toxic metabolite, as shown with the drug brivudine.^44^ In this regard, our data should be considered a guide, rather than a definitive conclusion, for prioritizing follow-up studies. Moreover, the quantitative assessment of the gut microbial ODM of etoposide showed that a metabolite with a single ODM is a primary metabolic product, whereas the qualitative assay detected two metabolites with *m/z* peaks corresponding to single and double ODM of etoposide. As we have shown with etoposide as a model drug, the significance of the gut microbial ODM of other ODM-positive drugs needs further investigation.

We identify seven gut bacteria that can *O*-demethylate etoposide, including three previously known ODMs of botanical compounds and four previously unknown for ODM activity. Whether these bacteria *O*-demethylate other ODM-positive drugs identified remains to be examined. The collection of 56 human gut bacteria tested for etoposide ODM in this study represents only a tiny fraction of the bacterial species (>1000)^45^^,^^46^ detected in the human gut microbiota, and additional gut bacterial species with ODM activity are likely exist. Notably, despite the significant gut microbial ODM of etoposide observed in mouse cecal suspensions or human stool samples, monocultures of the seven gut bacterial strains identified generated only trace amounts of M1. This low ODM activity in individual cultures may reflect the low expression of genes involved in etoposide ODM *in vitro* under our experimental conditions.

Several previous studies have examined the gut microbial metabolism of hundreds of drugs, analyzing samples similar to ours using HPLC–HRMS. Their tested drugs include some of the 35 ODM-positive drugs detected in our study. A study by Zimmermann et al.^5^ reported 18 ODM-positive drugs, and Javdan et al.^4^ reported 17 ODM-positive drugs. However, none of the shared drugs in these previous studies were found to yield notable gut microbial ODM under their test conditions. In the study by Zimmermann et al., 76 gut bacteria were individually tested for drug metabolism, and this collection included one (*E. hallii*) of the seven gut bacteria identified in our study to *O*-demethylate etoposide, perhaps suggesting an inability of *E. hallii* to *O*-demethylate those shared drugs. Alternatively, the variation in ODM activity of *E. hallii* may arise from methodological differences in screening for gut bacterial drug metabolism. Zimmermann et al. screened cultures of individual gut bacteria incubated with a pool of multiple drugs, whereas we tested individual drugs for gut bacterial ODM using a mouse cecal suspension. In the screen by Zimmermann et al., one drug in the pool could alter the physiology of the test bacterium and inhibit the metabolism of another, leading to an apparent absence of bacterial metabolism. In this context, our screen using a mouse cecal suspension may provide a more physiologically relevant context. Such methodological differences may underlie our identification of ODM-positive drugs, including some that overlapped with those examined by Zimmermann et al. but were not detected as ODM-positive in their screen.

Javdan et al. initially examined gut microbial drug metabolism using an *ex vivo* culture of a single human stool sample grown in a medium; however, they did not observe the ODM of 17 shared drugs, including etoposide. This result may be due to the absence or very low abundance of *O*-demethylating gut bacteria in the human stool sample used in their study or the suppression of gut bacterial ODM activity in their *ex vivo* medium. In a recent study by Wu et al.^25^, which was published during our manuscript preparation, a consortium of 30 gut bacterial species was examined for the metabolism of 127 G-protein-coupled receptor-targeted drugs, including seven drugs shared with our study (four ODM-negative drugs: cisapride, iloperidone, melatonin, and zafirlukast; three ODM-positive drugs: carvedilol, doxazosin, and trimethobenzamide); three drugs not tested in our study (sarpogrelate, SB756050, and trimebutine). Wu et al. confirmed our finding of doxazosin and trimethobenzamide and also identified another drug, SB756050, as a substrate of gut bacterial ODM. The bacterial community used in their study included two etoposide-*O*-demethylating bacteria identified in our study, *B. producta* and *E. callanderi*. While the authors have not examined the ODM activity of these two bacteria for their tested drugs, either or both of the bacteria are likely responsible for their observations, which remains to be determined.

Etoposide, the model drug for gut microbial ODM in this study, has been known to exhibit widely varying oral bioavailability among individuals, ranging from 25% to 97% (with an average of ~50%).^27^^,^^28^ However, such large inter-individual variability in etoposide oral bioavailability has been a cause of concern owing to the narrow therapeutic range of etoposide (0.5–3 μg/mL),^47^ its underlying sources have been unclear.^28^^,^^48^^,^^49^ In this study, we demonstrate that the depletion of the gut microbiota (via one-day antibiotic pretreatment) in mice results in a 1.9-fold increase in systemic etoposide exposure and a 1.8-fold increase in etoposide oral bioavailability, accompanied by a 3.7-fold decrease in the etoposide ODM product M1. Importantly, we did not observe these pharmacokinetic changes when etoposide was administered intravenously to antibiotic-treated mice. These results have strongly supported our conclusion that gut microbial ODM is a major elimination pathway for etoposide and the primary source of M1 production, suggesting that differential extents of gut microbial etoposide ODM likely contribute to inter-individual variability in the oral bioavailability of etoposide observed in humans.

Etoposide, as an anticancer agent, covalently stabilizes DNA-topoisomerase II, which mediates the re-ligation of the nicked DNA strands, consequently generating cytotoxic DNA strand breaks in cancer cells.^50^^,^^51^ On the other hand, etoposide treatment has been associated with an increased risk of secondary therapy-related acute myeloid leukemias (t-AMLs).^52^^,^^53^ Previous biochemical studies *in vitro* have shown that etoposide catechol (i.e., M1) is readily oxidized by myeloperoxidases (abundant in myeloid cells) to etoposide quinone and that both etoposide catechol and etoposide quinone are more potent than etoposide in inducing DNA strand breaks,^36^^,^^54-57^ implicating their genotoxic activity as a cause of the etoposide-associated development of t-AMLs. Our study has extended these previous *in vitro* findings in animal experiments, demonstrating that M1 is more genotoxic than the parent etoposide in mice, inducing more significant DNA damage in bone marrow cells. This result suggests that increased M1 production by the gut microbiota enriched with etoposide *O*-demethylating bacteria and, consequently, greater systemic exposure to genotoxic M1 may increase the risk of etoposide-associated t-AMLs. Our study illustrates a case of increased drug toxicity due to gut bacterial ODM, while the study by Wu et al.^25^ mentioned above reports that the respective *O*-demethylated metabolites of SB756050 and trimethobenzamide exhibit ~3-fold increased activity against their target GPCR protein, suggesting that gut bacterial ODM may alter drug efficacy and toxicity in a drug-specific manner.

In conclusion, we have presented gut bacterial ODM as a prevalent biotransformation reaction for drugs containing *O*-methylated aromatics and demonstrated its significant contribution to systemic drug exposure, with potential genotoxicity of the *O*-demethylated metabolite. Overall, our findings suggest that gut microbial ODM can be a determining factor in inter-individual variability in drug disposition, with altered drug efficacy and toxicity, and provide a rationale to investigate other ODM-positive drugs.

## Materials and methods

### Bacterial strains, human cell lines, chemicals, and animals

The bacterial strains used in this study (Table S2) were obtained from the BEI Resources, American Type Culture Collection (ATCC), or Deutsche Sammlung von Mikroorganismen und Zellkulturen (DSMZ) and were grown in the appropriate media as indicated in each experiment. MCF-7 (breast cancer; Cat. No. HTB-22), HeLa (cervical cancer; Cat. No. CCL-2), and Caco-2 (colorectal adenocarcinoma; Cat. No. HTB-37) cells were purchased from ATCC. The chemicals used in this study are listed in Table S5. C57BL6 male mice (8–10 weeks old) were purchased from Jackson Laboratory (Sacramento, CA) and housed in the animal facility at the University of Illinois at Chicago. The study protocol for the animal experiments was approved by the Institutional Review Board at the University of Illinois at Chicago (protocol number 20-179).

### Screening for drugs that are *O*-demethylated by the gut microbiota

Male 6–12 week-old C57BL/6 mice (Jackson RB15, Sacramento, CA) were euthanized by CO_2_ inhalation. The cecum was isolated, and its content was transferred to a conical centrifuge tube and weighed. The cecal contents were transferred to an anaerobic chamber (with a gas mixture of 5% H_2_, 5% CO_2_, and 90% N_2_; Anaerobe Systems, Morgan Hill, CA, USA) and resuspended in sterile pre-reduced PBS (final concentration 100 mg/mL). The suspension was subsequently centrifuged at 100* *g for 5 min at room temperature to remove large particles. The supernatant was then transferred to a fresh tube and diluted (1:1) with sterile pre-reduced PBS. Drugs were then spiked into the cecal supernatant at a final concentration of 50 µM and incubated in the anaerobic chamber at 37 °C for 24 h. The incubation was terminated by adding ice-cold acetonitrile (1:1), and the mixture was vortexed. The samples were centrifuged at 16,100 g for 10 min at room temperature. The supernatant was then collected and filtered (4 mm nylon syringe filter; 0.2 µm pore size) and analyzed using high-resolution mass spectrometry. Three independent experiments were performed.

### High-resolution mass spectrometer (HRMS) analysis for gut microbial *O*-demethylation

Separations were performed on an Agilent 1290 ultra-performance liquid chromatography (UPLC) system (Palo Alto, CA, USA), with a mobile phase flow rate of 0.4 mL/min and a 5 µL injection volume. The metabolites were assayed using a Waters Acquity BEH C18 column (1.7 µm, 2.1 × 100 mm), where mobile phases A and B were 0.1% formic acid in ddH_2_O and acetonitrile, respectively. The initial conditions were 95:5 (A:B), held for 1 min, followed by a linear gradient to 20:80 at 26 min and then 5:95 at 28 min. Column re-equilibration was performed by returning to 95:5 (A:B) at 29 min and holding until 35 min. The mass analysis was obtained using an Agilent 6545 Q-TOF high-resolution accurate mass spectrometer (HRMS) with an ESI capillary voltage +3.5 kV, a nitrogen gas temperature of 325 °C, a drying gas flow rate of 8.0 L/min, a nebulizer gas pressure of 30 psi, a fragmentor voltage of 130 V, a skimmer voltage of 45 V, and an OCT RF voltage of 750 V. Mass data were collected from *m/z* 70–1000 at 3 spectra/sec, using collision energies of 10, 20, and 40 V. The mass accuracy was improved by continuously infusing Agilent Reference Mass Correction Solution (G1969-85001). Peak deconvolution, integration, alignment, and *t*-test statistical analysis were performed using MS-DIAL (v. 4.7), comparing three biological replicates of drug exposure to the cecum blank. A demethylated metabolite was considered to be detected if the metabolite met all of the following criteria: (a) there was a mass difference of a multiple of −14 from the parent drug signal; (b) the difference between the *m/z* observed and the theoretical *m/z* was no more than 20 ppm; (c) the observed *m/z* signal was at least ten-fold higher than the highest signal observed in the cecum blank at the same retention time in at least two of the triplicates. A compound was considered to undergo gut microbial ODM if at least one metabolite that met the criteria was detected.

### Measurement of the overall gut microbial metabolism of ODM-positive drugs

A mouse cecal suspension was prepared as described above, spiked with individual compounds at a final concentration of 100 µM, and incubated anaerobically at 37 °C for 24 h. The incubation was terminated by adding one volume of ice-cold acetonitrile and vortexed briefly. Samples were centrifuged at 16,000 g for 10 min at 10 °C, and the supernatant was filtered using a 0.22 µm centrifugal filter unit (Millipore Sigma) at 16,000 g for 10 min at 10 °C. The final samples were analyzed using an Agilent 1100 HPLC system coupled with a G1314A UV detector. For each run, 40 µL of a sample was injected and resolved on a C18 column (XTERRA MS C18; 4.6 × 250 mm, 5 µm, Waters) using the HPLC–UV protocols listed in Table S6.

### Etoposide metabolism by mouse cecal and small intestinal contents and human stools

*Etoposide metabolism by mouse cecal contents.* Mouse cecal suspension was prepared as described above and pre-incubated anaerobically with pre-reduced PBS (as a control) or antibiotics (vancomycin 0.1 mg/mL, neomycin 0.1 mg/mL, and/or ampicillin 0.1 mg/mL) for 3 h, followed by the addition of etoposide (25 μg/mL) and additional incubation for 4 h at 37 °C. For heat treatment, the mouse cecal suspension was boiled for 10 min and cooled to room temperature before adding etoposide. The incubation was stopped by adding ice-cold acetonitrile (1:1), and the mixture was analyzed by HPLC–UV.

*Etoposide metabolism by human stools.* The study protocol for human stool sample collection was approved by the Institutional Review Board at the University of Illinois at Chicago (protocol number 2018-0810). Freshly collected human stool samples from two healthy donors were transferred into an anaerobic chamber immediately and resuspended in pre-reduced PBS (100 mg/mL). To remove large particles, the suspension was centrifuged at 100 g for 5 min at 25 °C. The supernatant was transferred into a fresh tube and diluted 2-fold with pre-reduced PBS. Etoposide (100 µg/mL) was incubated with the stool suspension anaerobically at 37 °C for 4 h. The incubation was terminated by adding an equal volume of ice-cold acetonitrile and vortexing vigorously for 30 s. The samples were subsequently centrifuged at 16,100 g x 10 min at 4 °C, after which the supernatants were collected for HPLC/UV or LC–tandem mass spectrometry (MS/MS) analyses.

*Etoposide metabolism by mouse small intestinal contents.* C57BL/6 male mice (6‒12 weeks old; Jackson RB15, Sacramento, CA) were euthanized by CO_2_ inhalation. The small intestine (encompassing the jejunum and ileum) was resected, and its contents were washed off with 5 mL pre-reduced PBS in the anaerobic chamber. After large particles were removed via centrifugation (100 g × 5 min), the supernatant was transferred to a new tube, adjusted with pre-reduced PBS to an OD_600_ of 2, and incubated anaerobically with etoposide (25 µg/mL) at 37 °C for 6 h. For comparison, cecal contents from the same mice were collected and processed as described above. The cecal suspension was adjusted to an OD_600_ of 2 with pre-reduced PBS and incubated anaerobically with etoposide (25 µg/mL) for 6 h. Etoposide and M1 concentrations were measured using HPLC–MS/MS as described below.

*Measurement of etoposide and M1.* The samples were analyzed by using a 2695 HPLC system (Waters Corp., Milford, MA, USA) coupled with a 2487 UV detector (Waters). Typically, 40 µL of a sample was injected and resolved on a C18 column (XTERRA MS C18; 4.6 × 250 nm; 5 µm, Waters) using acetonitrile (solvent A) and water (solvent B) as the mobile phase with the following gradient: 0–30 min (15%–35% A), 30–35 min (35%–100% A), and 35–40 min (15% A). The eluates were monitored at 210 nm. For further verification of M1, the supernatant was also analyzed by HPLC‒MS/MS. An Agilent 1200 HPLC (Agilent, Santa Clara, CA, USA) instrument interfaced with an Applied Biosystems (Foster City, CA, USA) Qtrap 3200 using an electrospray ion source. The separation was performed on an XTerra MS C18 column (3.5 μm, 50 mm × 2.1 mm, i.d., Waters), and the mobile phase consisted of acetonitrile (solvent A) and 0.1 mM ammonium formate (solvent B) containing 0.1% formic acid. The HPLC system (Agilent 1200) was operated at an isocratic flow rate of 0.32 mL/min (A:B, 80:20). The total run time for a sample was 2.5 min each. The selected reaction monitoring transitions were *m/z* 606.2–229.2 for etoposide, *m/z* 592.2–229.2 for M1, and *m/z* 679.1–405.1 for the internal standard (teniposide). All the data were acquired and analyzed using the Analyst version 1.5 software (AB SCIEX, Framingham, MA, USA).

### Purification and structure determination of M1

M1 was mass-produced and purified as follows. Human stool samples (25 g) were resuspended in 500 mL of pre-reduced PBS containing 30 mg of etoposide and incubated anaerobically at 37 °C for 2 d. After 2 d of incubation, the mixture was extracted with 500 mL of an organic solvent (hexane:chloroform, 3:7) twice. The organic layer was collected and evaporated using a rotary evaporator. The dried extract was dissolved in 10 mL of methanol, subjected to solid-phase extraction (SPE) using an SPE C18 cartridge (Hypersep C18 5000 mg, Thermo), and eluted with 20% acetonitrile. M1 was purified using HPLC-UV system (Waters) equipped with a Microsorb 60-8, C18 Dynamax column (Agilent; 250 × 10 mm, 8 µm). The mobile phase consisted of acetonitrile (solvent A) and water (solvent B), and the following gradient was used: 0–20 min (15%–32% A), 26–33 min (100% A), and 33–40 min (15% A) with a flow rate of 5 mL/min. A peak at 21.5 min corresponding to M1 was collected, dried, and subjected to structure determination.

The LC–MS/MS analysis of etoposide, commercial etoposide catechol, and the purified M1 was conducted, respectively, using an ImpactII (Bruker) Qq-TOF mass spectrometer equipped with an electrospray ionization source operating in positive ionization mode connected to a reversed-phase C18 UHPLC column (Phenomenex Kinetex, 50 × 2.1 mm) in a Nexera X2 series UHPLC system (Shimadzu). The respective samples were separated with an initial 15% acetonitrile (+0.1% formic acid) isocratic step for 0.5 min followed by a gradient over 6 min from 15% to 100% acetonitrile at 0.5 mL/min. The scan range was between 50 and 1500 *m/z*, with the spectral rate set to 6.0 Hz. The auto MS/MS setting was used to collect MS2 spectra, collecting data for 5 precursor ions per duty cycle requiring an absolute threshold of 316 cts. Ions were actively excluded after collecting 3 spectra, reconsidering the precursor ions if the current intensity was 2.0x greater than the previous intensity. The software DataAnalysis (Bruker, version 4.4) was used for post-run calibration and analysis of the MS data. Proton (^1^H) NMR spectra were acquired for commercial etoposide catechol and the purified M1 using a Bruker AVII 900 MHz spectrometer with a 5 mm CPTCI cryoprobe. The respective compounds were dissolved in 175 μL of 99.9% DMSO-*d*_6_ with an additional drop of 99.96% D_2_O to remove all exchangeable proton signals from the NMR spectra. The spectra were calibrated to the DMSO-*d*_6_ solvent peak (δ_H_ 2.50).

### Identification of gut bacteria that *O*-demethylate etoposide

Individual bacteria were grown in appropriate media (Table S2). Bacterial cells harvested from the respective bacterial cultures by centrifugation (4,000* *g × 15 min) were resuspended in MGYC broth (M9 medium containing 0.5% glucose, 0.05% yeast extract, and 0.1% cysteine) at an OD_600_ of 2. The respective bacterial resuspension (1 mL) was incubated anaerobically with etoposide (final concentration of 10 µg/mL) at 37 °C. After 24 h of incubation, the internal standard (100 µL of 1 µg/mL teniposide) was added, and the mixture was extracted with 2 mL of ethyl acetate. The organic phase was dried in a speed vac, reconstituted in 100 µL of 50% acetonitrile, and analyzed by LC–MS/MS as described in the section on “etoposide metabolism” above.

### Mutant construction in *E. limosum* and test for etoposide *O*-demethylation

Mutants were constructed in the wild-type *E. limosum* ATCC 8486, as described previously.^32^ All the primers used are listed in Table S4. To test the mutant and wild-type strains for etoposide ODM, the cells were harvested from the respective overnight cultures grown in BHIc media supplemented with clarithromycin (1 µg/mL) and resuspended in gut microbiota medium (GMM) at OD_600_ of 0.5. The respective resuspension was incubated anaerobically with etoposide (50  µM) at 37 °C. An aliquot (300 µL) was sampled at 0, 2, 6, 8, and 24 h and mixed with 900 µL of ethyl acetate (containing 1 µg/mL of teniposide as the internal standard). After vortexing vigorously, the mixtures were centrifuged at 16,000 g for 10 min, and the upper organic phase was transferred to a fresh tube and dried using a speed vac. The dried residue was dissolved in 300 µL acetonitrile, and M1 concentration was determined using LC–MS/MS.

### Etoposide and M1 cytotoxicity assay

To assess the cytotoxicity of M1 compared to etoposide, sulforhodamine B assay was used as described previously,^58^ with slight modifications. Briefly, MCF-7 (breast cancer) and HeLa (cervical cancer) cells were grown in Dulbecco's modified Eagle's medium (DMEM; Gibco, Cat. No. 11995073) supplemented with 10% fetal bovine serum (GeminiBio, Cat. No. 100-106) and cells (6 × 10^3^ cells/well) seeded in a 48-well tissue culture plate. The cells were allowed to attach overnight and then treated with increasing concentrations of etoposide, commercial etoposide catechol, and purified M1. After 24 h of treatment, the medium was removed, and the cells were fixed using 10% formalin at room temperature for 30 min, washed, dried, and stained with sulforhodamine B (0.04% w/v) at room temperature for 1 h. Unbound SRB was washed with 1% (v/v) acetic acid. Bound sulforhodamine B was solubilized with 10 mM Tris base solution, and the absorbance was measured using a Synergy Mx Plate reader (BioTek) at 540 nm. The viability of cells treated with respective compounds is expressed as a percentage relative to that of untreated controls. Three independent experiments were conducted, and the IC_50_ values of the respective compounds were determined using GraphPad Prism 9.

### Etoposide and M1 genotoxicity: comet assay

The mice were injected with etoposide, M1 (5 mg/kg, i.v.) or vehicle control and sacrificed 1 h after dosing. Bone marrow cells were collected as described previously,^59^ with slight modifications. Bone marrow cells were extracted from one femur of each mouse into 5 mL of DMEM using a 22-gauge needle as follows. The femur was cleaned by removing the surrounding muscles and tissues using sterile forceps and scissors, and the femur was cut at both ends. The needle was then inserted into the bone marrow canal, and the cells were flushed using a 5 mL syringe containing DMEM solution. The bone marrow cells were subsequently centrifuged, washed, and resuspended in ice-cold PBS (Ca^2+^ and Mg^2+^ free). A single-cell gel electrophoresis comet assay was performed using a Comet SCGE assay kit (Enzo Life Sciences, NY, USA) according to the manufacturer's protocol. Briefly, bone marrow cells were resuspended in ice-cold PBS at a cell density of 1 × 10^5^ cells/mL. Then, 50 µL of cells were mixed with 500 μL of low-melting-point agarose and transferred to 75 μL aliquots on pre-warmed slides. The slides were kept at 4 °C to allow the gel to solidify and immersed in pre-chilled lysis solution for 1 h, followed by an alkaline solution (pH > 13) for an additional 1 h. Electrophoresis was performed at 20 V in Tris-borate-EDTA buffer (89 mM Tris, 89 mM boric acid, and 2 mM EDTA; pH 8.3) for 15 min. Comets were stained with CYGREEN dye solution for 30 min, rinsed, and dried for visualization. Comet images were acquired using a 20× objective on a Nikon Eclipse E600 microscope equipped with a DS-Ri1 digital camera and NIS Elements Software (Nikon Instruments). The tail moments were analyzed automatically using CometScore software (TriTek Corp., Sumerduck, VA), which determines the tail moments of 125 cells per sample.

### Caco-2 transport assay

Transwell® (PET membrane, 0.4 µm pore size, 1.12 cm^2^) individual inserts were purchased from Corning Life Science (MA, USA). Caco-2 cells were grown in 75 cm^2^ flasks in DMEM containing 10% fetal bovine serum in an incubator at 37 °C with 5% CO_2_ and 90% relative humidity. The cells were subcultured at 80% confluency by treatment with trypsin-EDTA (Gibco, Cat. No. 25200072). The Caco-2 cells were seeded onto the insert at a density of 1.5 × 10^4^ cells per insert. The medium was changed every other day thereafter, with an apical volume of 0.4 mL and a basolateral volume of 0.6 mL. The integrity of the monolayer was confirmed by measuring the transepithelial electrical resistance (TEER) of each cell insert before and after the transport experiment, and monolayers with TEER values above 300 Ω·cm^2^ were used. In addition to TEER, an impermeable fluorescent dye, lucifer yellow, was added to the apical side of each well and measured at the end of the experiment in the basolateral wells to examine membrane integrity. A transport study across Caco-2 cell monolayers was performed 18 d post-seeding. All transport studies were conducted with Hank's balanced salt solution (HBSS) supplemented with 25 mM HEPES. The cell monolayers were washed twice and preincubated for 30 min at 37 °C in HBSS, after which the TEER value was measured. The experiments were initiated by replacing the apical side of the cells with 0.4 mL of a test compound (10 µM) in HBSS. The cells were incubated at 37 °C on a rocker operated at 40 rpm throughout the experiment. The samples were collected at 0, 25, 50, 75, and 100 min for further analysis. The TEER value was measured at the end of the experiment to determine whether the integrity of the cell monolayers was maintained during the experiment. Compound concentration was measured by LC–MS/MS analysis and the apparent permeability (*P*_*app*_) was calculated using the following equation: *P*_*app*_ = (dQ/dt) × (1/C_0_) x (1/A), where dQ/dt is the rate of appearance on the basolateral side of the compartment, A is the surface area of the membrane (cm^2^), and C_0_ is the initial concentration of the test compound on the apical side.

### Etoposide and M1 metabolism in mouse liver microsomes

Mouse hepatic microsomes (Corning Life Sciences, Durham, NC, USA; 0.5 mg/mL) were incubated with etoposide, M1, and darunavir (1 µM) in a reaction mixture (1 mM NADP^+^, 5 mM MgCl_2_, 0.2 U/L isocitrate dehydrogenase, and 5 mM isocitric acid) at 37 °C with continuous shaking at 300 rpm. Darunavir was used as a positive control for the validity of the assay. The reaction was stopped at different time points (0, 5, 15, 30, and 60 min) by adding two volumes of ice-cold acetonitrile containing an internal standard (2 µM). The mixture was then centrifuged (12,000 g × 10 min at 4 °C), and the supernatant was stored at −20 °C until LC‒MS/MS analysis was performed to determine the fraction of remaining test compounds. The internal standards used were teniposide (for etoposide and M1) and phenytoin (for darunavir).

### Antibiotic treatment

To determine the effects of one-day antibiotic treatment on hepatic and small intestinal gene expression, the mice were administered an antibiotic cocktail (0.5 g/L vancomycin, 0.1 g/L polymyxin B) in their drinking water for 24 h. After 24 h of antibiotic treatment, the mice were fasted for 3 h and sacrificed. Liver, small intestine, cecum, and fecal samples (before and after antibiotic treatment) were collected, snap-frozen, and stored at −80 °C until further processing and analysis.

### Quantification of 16S rRNA gene copies in mouse cecal contents and fecal samples

DNA was isolated from mouse cecal contents and fecal samples, respectively, using the QIAamp PowerFecal Pro DNA Kit (Qiagen) according to the manufacturer's instructions and was used as a template in quantitative PCR (qPCR) to amplify the 16S rRNA genes. qPCR was performed with previously validated TaqMan probes (Table S4)^60^^,^^61^ on a StepOnePlus Real-Time PCR System (Applied Biosystems). Reactions were carried out in 96-well plates using the FastStart Universal Probe Master Mix (Roche) to quantify the 16S rRNA gene copy number. Each reaction was performed in duplicate in a final volume of 10 µL with 0.5 µM final concentration of each primer and a 0.25 µM final concentration of probe. Amplifications were performed with the following thermal cycling conditions: 1 cycle at 50 °C for 2 min; 1 cycle at 95 °C for 10 min; and 40 cycles of 95 °C for 15 s and 60 °C for 1 min.

### Quantitative real-time (qRT)-PCR for hepatic and small intestinal genes

Tissue samples (liver, jejunum, and ileum) were collected from control and antibiotic-treated mice. Total RNA was isolated from tissues using Trizol (Life Technologies, Carlsbad, CA, USA) and used as the template for the synthesis of complementary DNA using high-capacity cDNA archive kit (Applied Biosystems). Using the cDNA as a template, qRT-PCR was performed with the primers from Integrated DNA Technologies (San Diego, CA, USA) (Table S4). The fold change in mRNA levels was determined after normalization of the gene expression levels to those of Gapdh using the 2^−^^ΔΔCt^ method.^62^

### Gut permeability testing using FITC-dextran

The integrity of the intestinal tight junction barrier was determined by measuring the permeability of fluorescein isothiocyanate (FITC)-dextran.^63^ Briefly, C57BL/6J male mice (8-week old) were treated with the antibiotic cocktail in drinking water or regular water (5 mice/group) for 24 h, fasted for 3 h, and then dosed with 600 mg/kg (80 mg/mL) FITC-dextran (4 kDa, Sigma). Blood samples (~30 µL) were collected from saphenous veins into K3-EDTA tubes before (0 min) and 30, 60, and 240 min after FITC-dextran administration. The blood was centrifuged (12,000 g, 10 min, 4 °C) for plasma collection. The plasma samples were diluted 5-fold with PBS, and fluorescence was measured in duplicate using a Synergy Mx Plate reader (BioTek, Winooski, VT, USA) (excitation at 485 nm and emission at 535 nm) in black 96-well plates. Concentrations were calculated based on a standard curve.

### Etoposide and M1 pharmacokinetics

Animals had free access to water and food throughout the experiment unless otherwise indicated. For pharmacokinetic studies, the mice were divided into two groups (*n* = 18–20/group) for intravenous (i.v.) and oral dosing, respectively. Each route of administration had two groups with or without antibiotic treatment (*n* = 9–10/group). After 24 h of antibiotic treatment, the antibiotic water was replaced with regular water, and the food was removed from all the groups. To reduce variability in intestinal absorption, all the mice were fasted for 3 h before drug administration. For oral administration, Etoposide Injection USP (Accord Healthcare Inc., 20 mg/mL) was diluted 6-fold with a 0.9% NaCl solution, and a total volume of 6 μL/g (20 mg/kg) body weight was administered by oral gavage, using a curved blunt-ended needle to the mice. For i.v. dosing, etoposide injection USP (20 mg/mL) was diluted 10-fold with 0.9% NaCl, and a total volume of 5 μL/g body weight (10 mg/kg) was administered via the tail vein. After dosing, blood (~15 μL) was collected at multiple time points for each group through a saphenous vein in K3-EDTA tubes and stored at −20 °C until further analysis. The time points for the oral dosing groups were 0.25, 0.5, 1, 1.5, 3, 5, 7, and 9 h post-dosing, while for the i.v. dosing groups, the time points were 0.083, 0.25, 0.5, 1, 1.5, 3, 5, and 7 h post-dosing.

Etoposide and M1 concentrations in mouse blood samples were measured by using LC–MS/MS analysis as previously described with some modifications.^39^ Briefly, mouse blood samples (10 μL) from each time point were diluted to 100 μL with drug-free mouse blood. After adding 100 μL of the internal standard (teniposide, 500 ng/mL in acetonitrile) to each sample, the mixture was extracted with 2 mL of chloroform in a glass tube, vortexed thoroughly for 1 min, and centrifuged at 4000 g for 5 min. After centrifugation, the organic layer was transferred into a new glass tube and dried using a constant stream of N_2_ gas. The dried residue was reconstituted with methanol (50 μL for oral samples and 100 μL for i.v. samples) and analyzed using LC–MS/MS in positive ionization mode (Qtrap 5500; Applied Biosystems). The separation was performed on an XTerra MS C18 column (3.5 μm, 50 mm × 2.1 mm; Waters), and the mobile phase consisted of acetonitrile (solvent A) and 0.1 mM ammonium formate (solvent B) containing 0.1% formic acid. The HPLC system (Agilent 1200) was operated at an isocratic flow rate of 0.32 mL/min (A:B, 8:2). The total run time for a sample was 2.5 min. The selected reaction monitoring transitions were *m/z* 606.2–229.2 for etoposide, *m/z* 592.2–229.2 for M1, and *m/z* 679.1–405.1 for the internal standard (teniposide). All the data were acquired and analyzed using the Analyst version 1.5 software (AB SCIEX, Framingham, MA, USA). Data were analyzed using non-compartmental analysis (NCA) via WinNonlin software (Pharsight Corp., Version 5.0.1.).

### Statistical analysis

Comparisons between groups were made using the Student's *t*-test or one-way ANOVA using GraphPad Prism 9. A *p*-value less than 0.05 was considered significant.

## Supplementary Material

Revision_Supplemental Table 1.xlsx Revision_Supplemental Table 1.xlsx

(Unmarked)_Revision_Supplemental Figures (SF1–6)_Tables (TS2–6).docx (Unmarked)_Revision_Supplemental Figures (SF1-6)_Tables (TS2-6).docx

## Data Availability

All data are available in the main text and the supplemental tables. Any additional information to reanalyze the data and the bacterial mutant strains constructed in this study will be available upon request.
