# Supplementary material for: Gut bacterial O-demethylation modulates systemic exposure to oral etoposide
Source: Gut Microbes. 2026 Feb 13;18(1):2628358. doi: 10.1080/19490976.2026.2628358 (PMC12915777; doi:10.1080/19490976.2026.2628358)
Supplement: (Unmarked)_Revision_Supplemental Figures (SF1–6)_Tables (TS2–6).docx — (Unmarked)_Revision_Supplemental Figures (SF1-6)_Tables (TS2-6).docx [file KGMI_A_2628358_SM8245.docx]

**Supplemental Figures and Tables**

Figure S1A (35 ODM-positive drugs)


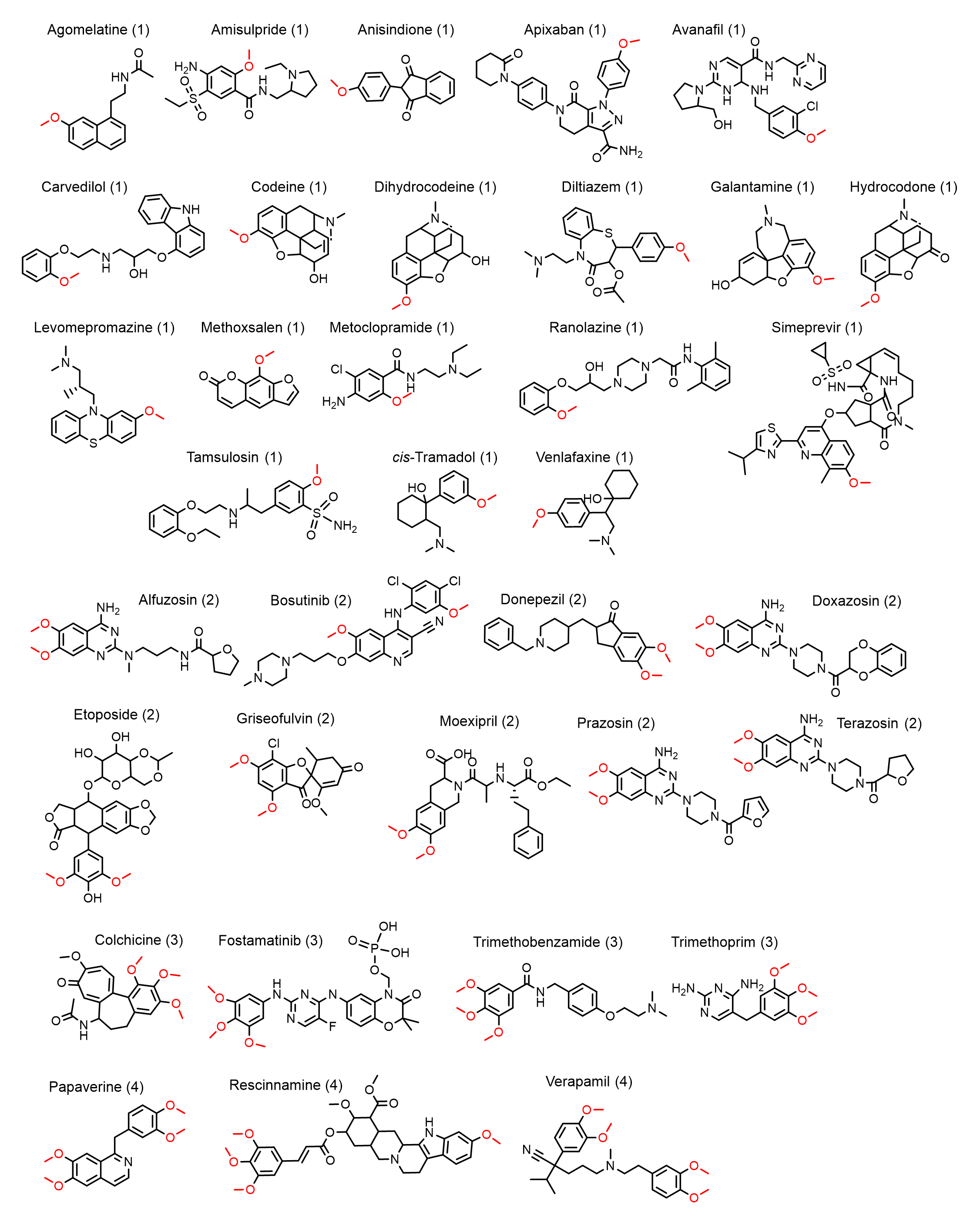


Figure S1B (29 ODM-negative drugs)


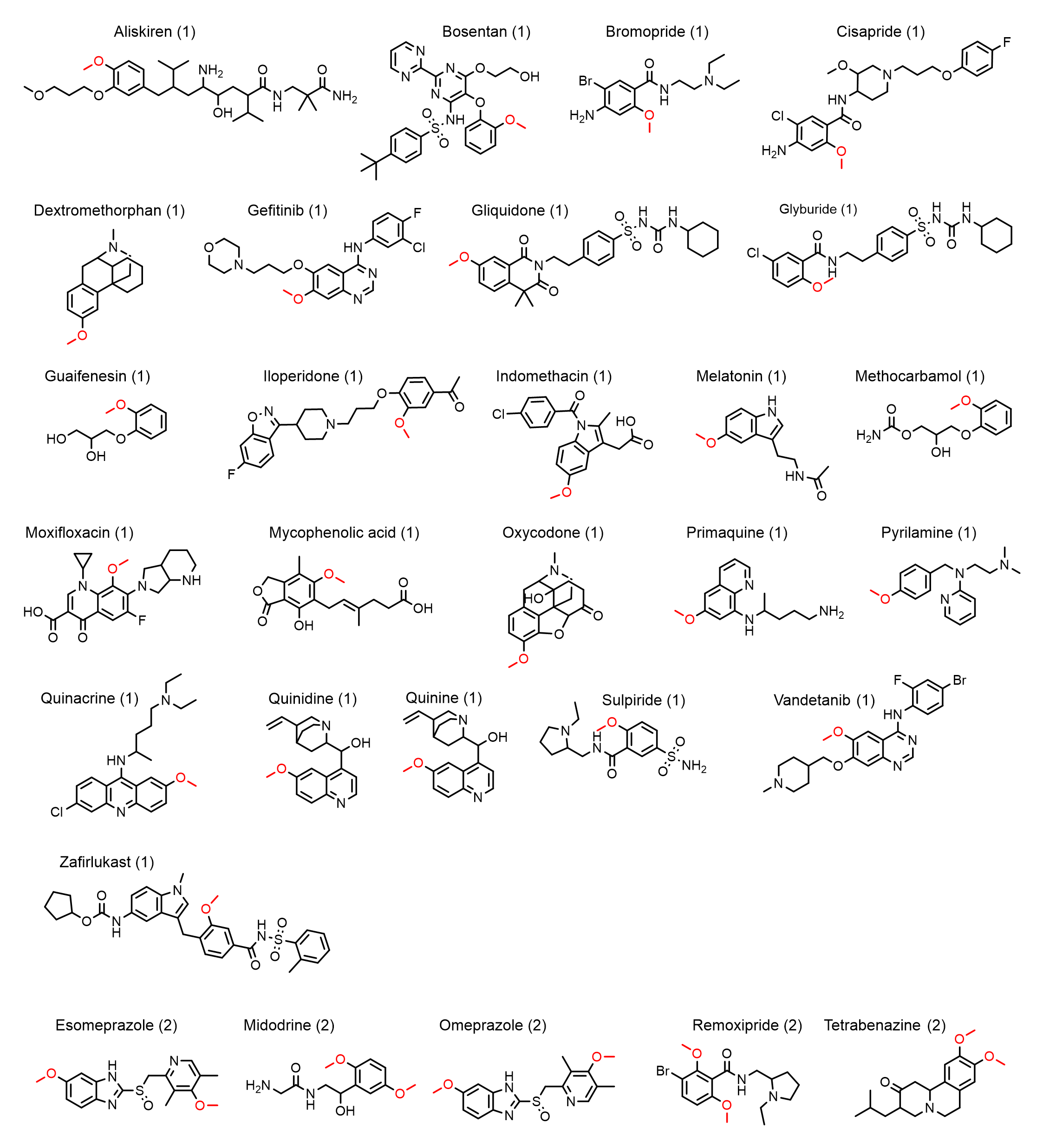


Figure S1C (two positive control compounds)


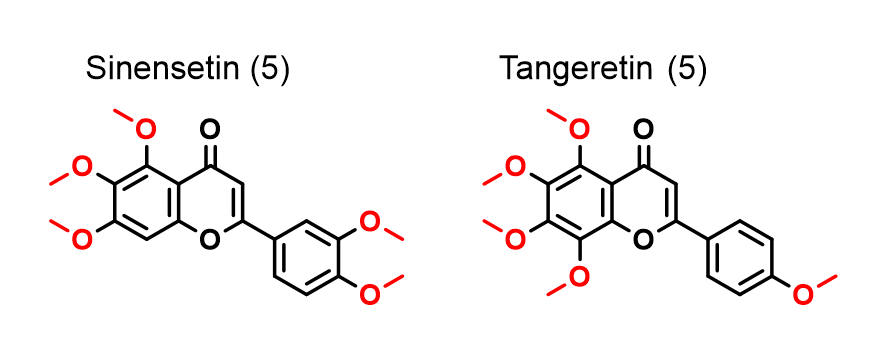


**Figure S1.** Chemical structures of compounds (64 drugs and two controls) tested for gut microbial *O*-demethylation. Parenthesis next to chemical names indicates the number of potential *O*-demethylation sites shown in red in chemical structures.


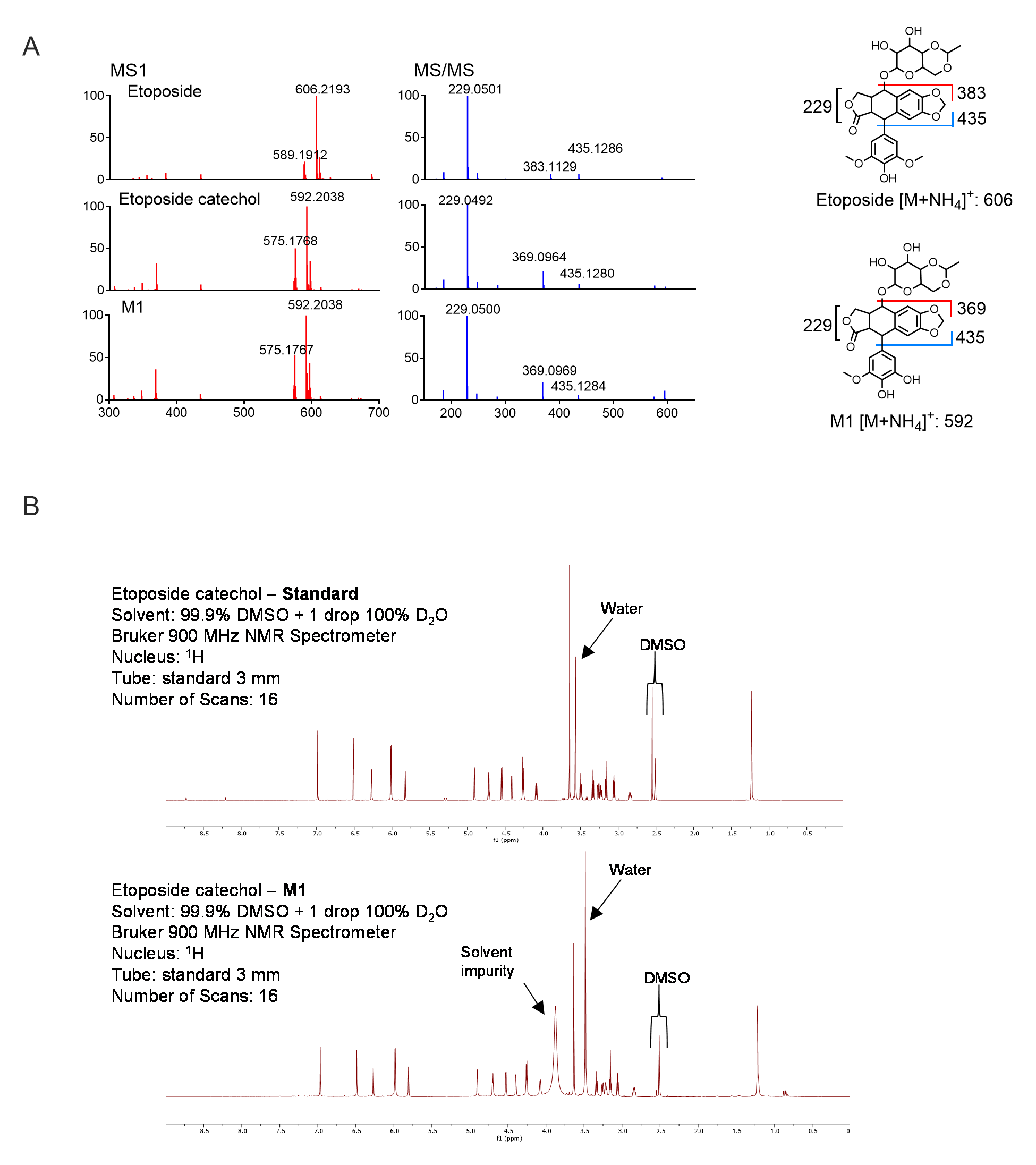


**Figure S2.** Structure determination of M1 as etoposide catechol. (A) The MS/MS fragmentation patterns of etoposide, etoposide catechol (purchased), and purified M1 were obtained by using LC-QTOF-MS/MS. (B) ^1^H-NMR spectra of etoposide catechol and purified M1.

**
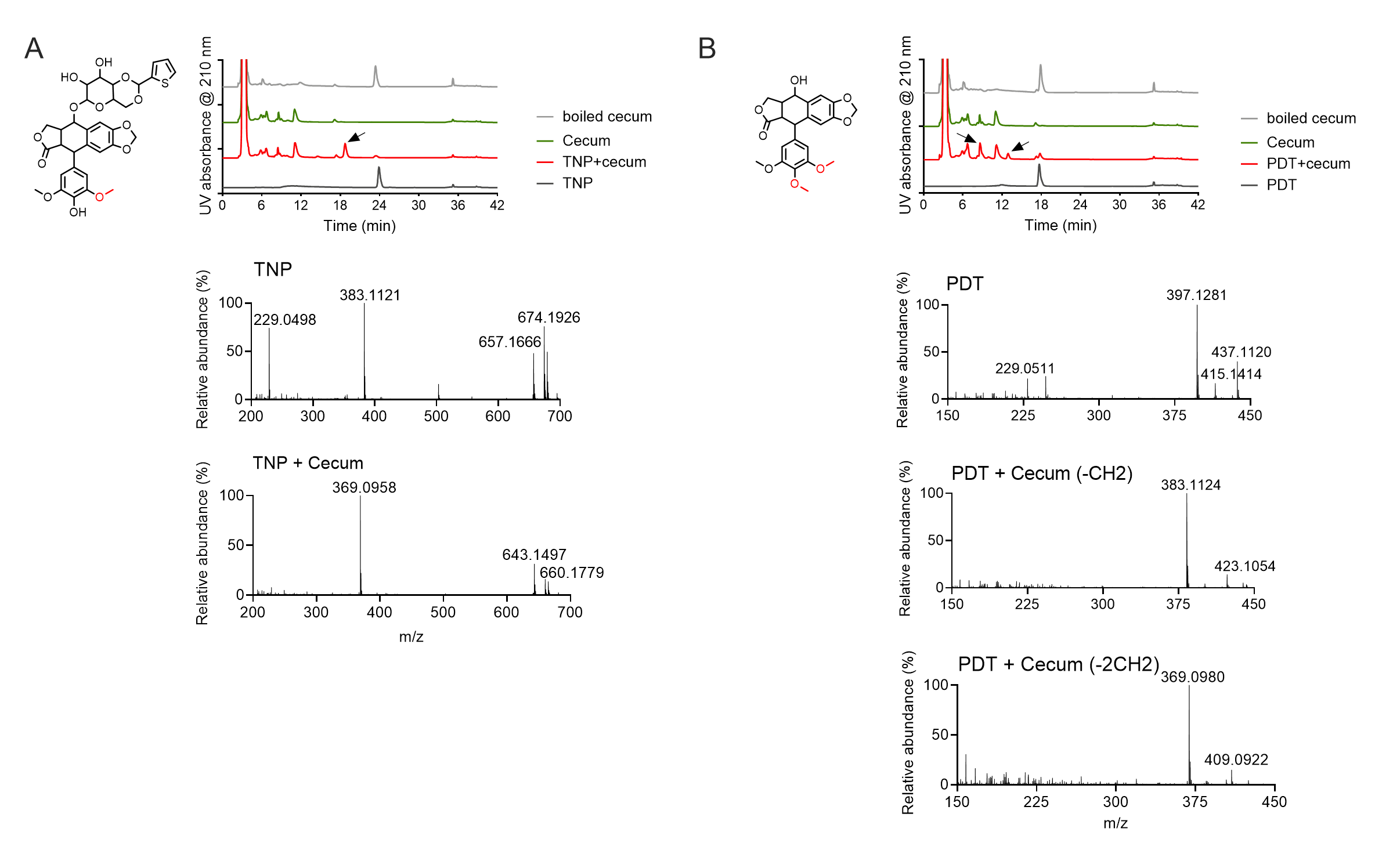
**

**Figure S3.** Teniposide and podophyllotoxin, structural analogs of etoposide, are also subject to gut microbial *O*-demethylation. Teniposide (TNP) and podophyllotoxin (PDT) at 100 μg/mL were incubated anaerobically in mouse cecum suspension (50 mg/mL) for 24 h, followed by HPLC-UV and LC-QTOF-MS/MS analysis. Arrows indicate *O*-demethylation products. (*n*=1)

**
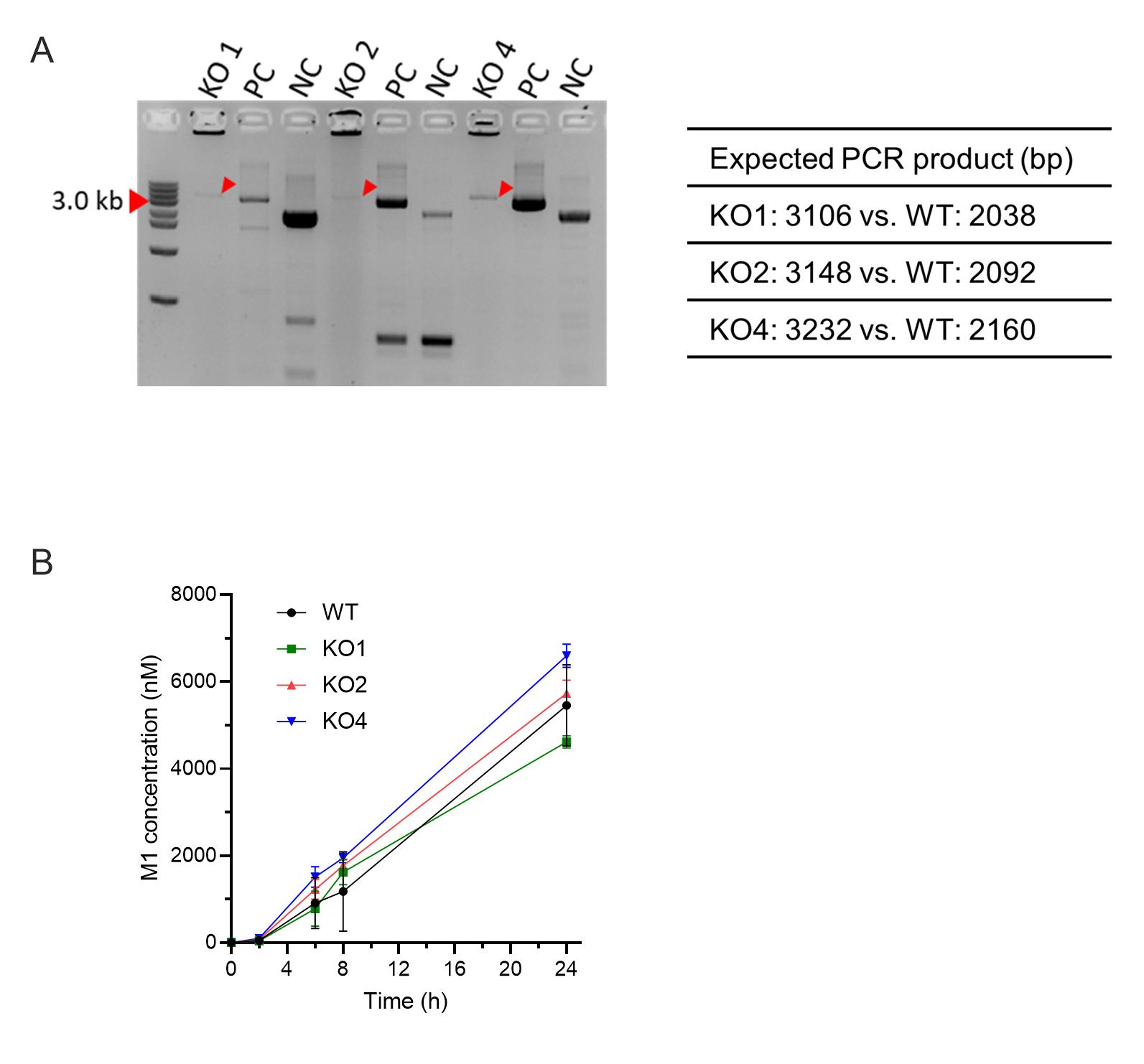
**

**Figure S4.** Three *E. limosum* mutants exhibit wild-type levels of M1 production.

(A) PCR verification of mutants constructed. Lane 1 (from the left): DNA ladder; Template in PCR for KO1 (lane 2), KO2 (lane 5), and KO4 (lane 8) were cells from a single colony inoculated to prepare cultures for purification of genomic DNA; Template in PCR for PC (lane 3, lane 6, and lane 9) were respective genomic DNAs isolated from KO1, KO2, and KO3 cultures; Template in PCR for NC (lane 4, lane 7, and lane 10) was genomic DNA isolated from wild-type *E. limosum* ATCC 8486. (B) Cells from overnight cultures of respective strains grown in Gut Microbiota Medium (GMM) were harvested and resuspended in fresh GMM at an optical density of 0.5 at 600 nm. Respective cell resuspension was incubated with etoposide (50 µM), and an aliquot was sampled at the indicated time points and analyzed for M1 production using UPLC-MS. Shown are the results of two independent experiments (*n*=2). WT (wild-type *E. limosum* ATCC 8486), KO1 (ΔB2M23_RS01060), KO2 (ΔB2M23_RS07870), KO3 (ΔB2M23_RS15135), and KO4 (ΔB2M23_RS16860).

**
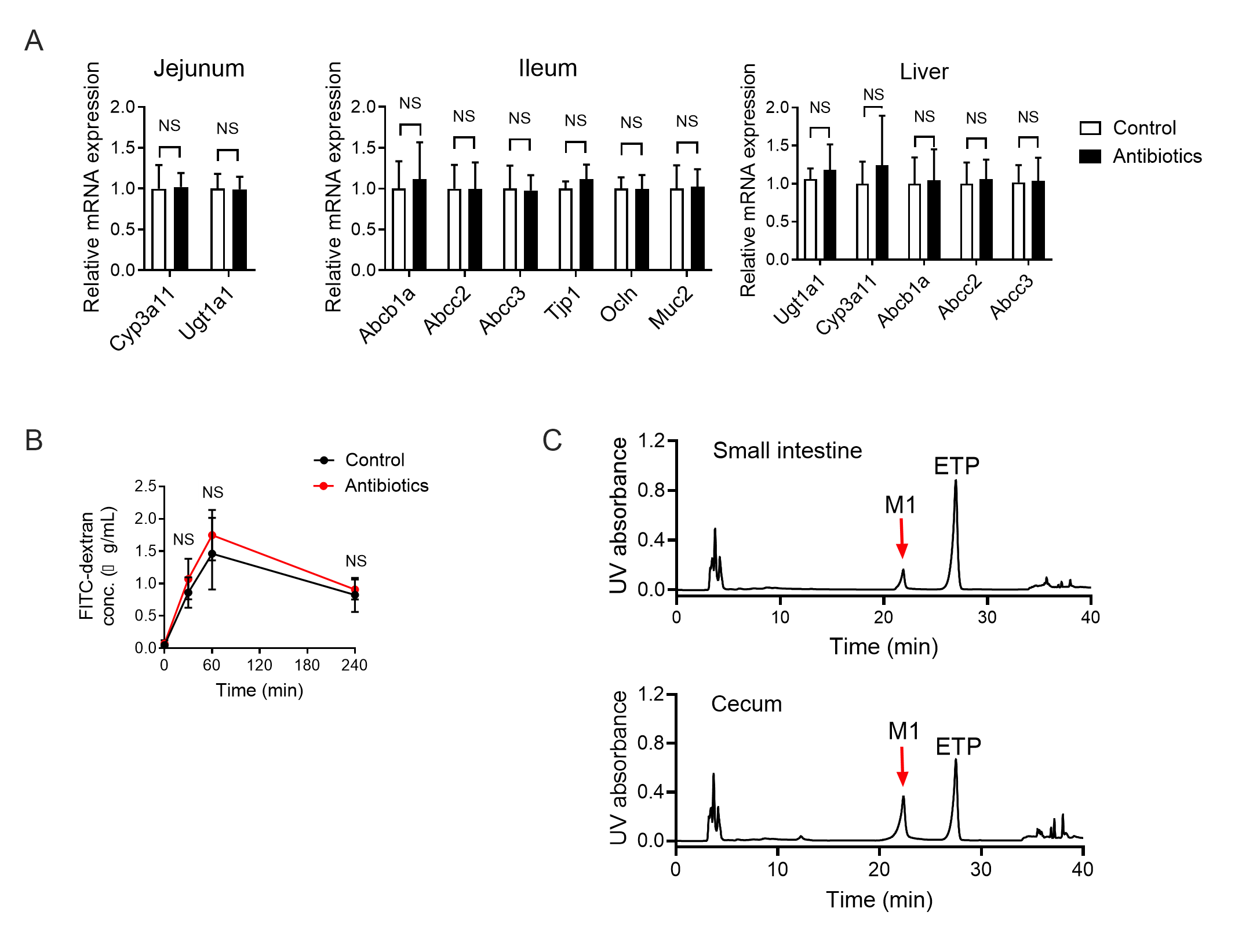
**

**Figure S5**. (A) C57BL6/J mice were pretreated with antibiotic-containing water (vancomycin, 0.5 g/L and polymyxin B, 0.1 g/L; or regular water as a control). After 24 h, antibiotic treatment was stopped, and mice were fasted for 3 h and sacrificed. mRNA expression levels of multiple genes in the jejunum, ileum, and liver tissues were determined using quantitative real-time PCR performed in triplicate (*n*=3). The data are represented as mean ± SD. (B) After 24 h of antibiotic treatment, FITC-dextran (600 mg/kg) was orally administered (*n*=5 mice/group), followed by blood collection at multiple time points. Plasma FITC-dextran concentration was measured using a plate reader. NS, not significant. The data are represented as mean ± SD. (C) Etoposide (25 μg/mL) was incubated for 6 h with either the small intestinal or cecal suspensions, normalized to an OD_600_ of 2. The reaction mixtures were analyzed by HPLC-UV, and M1 was further verified by LC-MS/MS. Shown is a representative of two or three independent experiments.

Table S2. List of 56 gut bacteria tested for the *O*-demethylation of etoposide into M1

| **No.** | **Phylum** | **Bacterium** | **Source (strain No.)** | **Growth medium** | **M1 production** |
| --- | --- | --- | --- | --- | --- |
| 1 | Actinomycetota | *Atopobium fossor* | DSMZ (DSM 15642) | BHI+ | No |
| 2 |  | *Bifidobacterium adolescentis* | BEI resources (HM-633) | YCFA | No |
| 3 |  | *Bifidobacterium angulatum* | BEI resources (HM-1189) | YCFA | No |
| 4 |  | *Bifidobacterium breve* | BEI resources (HM-856) | YCFA | No |
| 5 |  | *Bifidobacterium longum* | BEI resources (HM-845) | YCFA | No |
| 6 | Bacillota | *Agathobacter rectalis* | DSMZ (DSM 17629) | YCFA, BHI+ | No |
| 7 |  | *Anaerostipes sp.* | BEI resources (HM-220) | YCFA | No |
| 8 |  | *Blautia producta*^b^ | DSMZ (DSM 3507) | YCFA, BHI+ | Yes |
| 9 |  | *Clostridium aldenense* | BEI resources (HM-307) | BHI | No |
| 10 |  | *Clostridium cadaveris* | BEI resources (HM-1039) | YCFA | No |
| 11 |  | *Clostridium citroniae* | BEI resources (HM-315) | BHI | No |
| 12 |  | *Clostridium clostridioforme* | BEI resources (HM-306) | YCFA | No |
| 13 |  | *Clostridium innocuum* | BEI resources (HM-173) | BHI | No |
| 14 |  | *Coprobacillus sp.* | BEI resources (HM-176) | YCFA | No |
| 15 |  | *Coprococcus sp.* | BEI resources (HM-794) | YCFA | No |
| 16 |  | *Dorea formicigenerans* | BEI resources (HM-300) | BHI | No |
| 17 |  | *Enterococcus casseliflavus* | DSMZ (DSM 20680) | BHI+ | No |
| 18 |  | *Enterococcus gallinarum* | DSMZ (DSM 24841) | BHI+ | No |
| 19 |  | *Enterococcus faecalis* OG1RF^b^ | ATCC (47077) | BHI | No |
| 20 |  | *Enterococcus faecium* | DSMZ (DSM 20477) | BHI+ | No |
| 21 |  | *Enterococcus lactis* | DSMZ (DSM 23655) | BHI+ | No |
| 22 |  | *Enterococcus ventriosum* | DSMZ (DSM 3988) | BHI+ | No |
| 23 |  | *Erysipelotrichaceae sp 6* | BEI resources (HM-180) | YCFA | No |
| 24 |  | ***Eubacterium aggregans*** | DSMZ (DSM 12183) | YCFA, BHI+ | **Yes** |
| 25 |  | *Eubacterium barkeri* | DSMZ (DSM 1223) | BHI+ | No |
| 26 |  | *Eubacterium callanderi*^b^ | DSMZ (DSM 3662) | YCFA, BHI+ | Yes |
| 27 |  | ***Eubacterium eligens*** | DSMZ (DSM 3376) | YCFA, BHI+ | **Yes** |
| 28 |  | ***Eubacterium hallii*** | DSMZ (DSM 3353) | YCFA, BHI+ | **Yes** |
| 29 |  | *Eubacterium limosum*^b^ | ATCC (8486) | YCFA, BHI+ | Yes |
| 30 |  | ***Eubacterium ramulus*** | DSMZ (DSM 15684) | YCFA | **Yes** |
| 31 |  | *Faecalibacterium prausnitzii* | DSMZ (DSM 17677) | YCFA | No |
| 32 |  | *Granulicatella adiacens* | BEI resources (HM-1047) | BHI | No |
| 33 |  | *Lachnospiraceae sp.* | BEI resources (HM-1043) | YCFA | No |
| 34 |  | *Lactobacillus rhamnosus* | BEI resources (HM-106) | YCFA | No |
| 35 |  | *Megasphera elsdenii* | DSMZ (DSM 20460) | BHI+ | No |
| 36 |  | *Megasphera micronuciformis* | DSMZ (DSM 17226) | BHI+ | No |
| 37 |  | *Oscillibacter sp.* | BEI resources (HM-1030) | BHI | No |
| 38 |  | *Peptostreptococcus anaerobius* | DSMZ (DSM 20357) | BHI+ | No |
| 39 |  | *Ruminococcaceae sp.* D16 | BEI resources (HM-79) | BHI | No |
| 40 |  | *Ruminococcus gnavus* | BEI resources (HM-1056) | BHI | No |
| 41 |  | *Weissella cibaria* | BEI resources (HM-1200) | YCFA | No |
| 42 | Bacteroidota | *Bacteroides cellulosilyticus* | BEI resources (HM-726) | YCFA | No |
| 43 |  | *Bacteroides dorei* | DSMZ (DSM 17855) | YCFA | No |
| 44 |  | *Bacteroides eggerthii* | BEI resources (HM-210) | YCFA | No |
| 45 |  | *Bacteroides finegoldii* | BEI resources (HM-727) | YCFA | No |
| 46 |  | *Bacteroides fragilis* | BEI resources (HM-709) | YCFA | No |
| 47 |  | *Bacteroides ovatus* | BEI resources (HM-222) | YCFA | No |
| 48 |  | *Bacteroides stercoris* | BEI resources (HM-1036) | YCFA | No |
| 49 |  | *Bacteroides thetaiotaomicron* | ATCC (29148) | YCFA | No |
| 50 |  | *Bacteroides vulgatus* | BEI resources (HM-720) | YCFA | No |
| 51 |  | *Parabacteroides distasonis* | BEI resources (HM-169) | YCFA | No |
| 52 |  | *Parabacteroides johnsonii* | BEI resources (HM-731) | YCFA | No |
| 53 |  | *Parabacteroides merdae* | BEI resources (HM-729) | YCFA | No |
| 54 | Fusobacteriota | *Fusobacterium nucleatum* | BEI resources (HM-992) | YCFA | No |
| 55 | Pseudomonadota | *Escherichia coli K-12* | Jeong Lab (MG1655) | YCFA | No |
| 56 |  | *Sutterella wadsworthensis* | DSMZ (DSM 14016) | BHI+ | No |

^a^BHI+: BHI supplemented with L-cysteine-HCl (0.05% w/v), vitamin K (1 μg/mL), and hemin (5 μg/mL).

^b^Gut bacteria previously known to have *O*-demethylating activity and found to *O*-demethylate etoposide in this study.

In bold: Gut bacteria previously unknown to have *O*-demethylating activity and found to *O*-demethylate etoposide in this study.

Table S3. Putative methyltransferase-I homologs in *E. limosum*

| MT-I in *E. callaneri* KIST612  (amino acids: aa) | MT-I homologs in *E. limosum* ATCC 8486  (aa; % coverage; % identity) |
| --- | --- |
| ELI_0483 (478) | 8 homologs:  - B2M23_RS12900 (478; 100; 53.8)  - B2M23_RS07975 (478; 100; 52.7)  - B2M23_RS03430 (478; 100; 52.7)  - B2M23_RS18960 (479; 100; 52.6)  - B2M23_RS20080 (478; 100; 52.5)  - B2M23_RS20095 (480; 100; 52.5)  - B2M23_RS20100 (479; 100; 51.6)  - B2M23_RS20085 (481; 100; 51.6) |
| ELI_0486 (473) | 2 homologs:  - B2M23_RS03455 (474; 95.98; 30.6)  - B2M23_RS05475 (489; 93.66; 30.3) |
| ELI_0558 (475) | **B2M23_RS01060** (474; 100; **98.5**) 🡪 **KO1** |
| ELI_1591 (382) | **B2M23_RS15135** (382; 100; **98.4**) 🡪 KO3 |
| ELI_2003 (460) | **B2M23_RS16860** (460; 100; **97.8**) 🡪 **KO4** |
| ELI_2930 (401) | B2M23_RS15135 (412; 99; 30.3) |
| ELI_3711 (479) | **B2M23_RS07870** (479; 100; **97.9**) 🡪 **KO2** |

- tBLASTn searches were performed with respective MT-I proteins of *E. calladeri* as bait in the genome of *E. limosum* using the software Geneious Prime (version 2023.2).

- Genomes used: *Eubacterium callanderi* KIST612 (GenBank accession No.: CP002273.2) and *Eubacterium limosum* ATCC 8486 (GenBank accession No.: NZ_CP019962.1).

- Mutant construction was attempted for four genes in boldface in *E. limosum*, and three mutants were obtained: KO1, KO2, and KO4.

Table S4. List of plasmids and oligonucleotides used in this study.

| **Plasmids** | | Source or purpose |
| --- | --- | --- |
| pJIR-Cas9 |  | Shin *et al*. (2019) |
| pJET |  | Shin *et al*. (2019) |
| pJET-LR-KO1-sgRNA |  | This study |
| pJET-LR-KO2-sgRNA |  | This study |
| pJET-LR-KO3-sgRNA |  | This study |
| pJET-LR-KO4-sgRNA |  | This study |
| **Oligonucleotides** (from Integrated DNA technologies; 5′🡪3′) | | |
| Cyp3a11 | Cat#: Mm.PT.58.8020763 | qRT-PCR |
| Ugt1a1 | Cat#: Mm.PT.58.9514311 | qRT-PCR |
| Ocln | Cat#: Mm. PT.58.30118962 | qRT-PCR |
| Tjp1 | Cat#: Mm. PT.58.12952721 | qRT-PCR |
| Muc2 | Cat#: Mm. PT.58.29496069.g | qRT-PCR |
| Abcb1a | Cat#: Mm.PT.56a.33328663 | qRT-PCR |
| Abcc2 | Cat#: Mm. PT.58.8147009 | qRT-PCR |
| Abcc3 | Cat#: Mm. PT.58.28437920 | qRT-PCR |
| KO1-LA-F | CTCGAGTTTTTCAGCAAGATGTACATCTGTGTAGCGGCGA | To PCR-amplify the left region of B2M23_RS01060 |
| KO1-LA-R | AAACCACAAAACGAAGCCAGCATGGAGATT |  |
| KO1-erm-F | CTGGCTTCGTTTTGTGGTTTATTTACAAATTCGGC | To PCR-amplify the *ermB* resistance cassette |
| KO1-erm-R | CAATGGACCGTGTTTGCAAGCAGCAGATTACGC |  |
| KO1-RA-F | CTTGCAAACACGGTCCATTGCTTCTGGACT | To PCR-amplify the right region of B2M23_RS01060 |
| KO1-RA-R | AGGAGATCTTCTAGAAAGATTGGTGGTTTCCGTTACCTCG |  |
| KO1-sgOligo-F | TTGACAGCTAGCTCAGTCCTAGGTATAATACTAGTCCATGCTGGCTTCGTGTACCGTTTTAGAGCTAGAAATAGCAAGTT | To prepare template for KO1-sgRNA |
| KO1- sgOligo-R | AAAAAAAGCACCGACTCGGTGCCACTTTTTCAAGTTGATAACGGACTAGCCTTATTTTAACTTGCTATTTCTAGCTCTAA |  |
| KO1-NotI-XhoI-sgRNA-F | ATAGGGAGAGCGGCCTTGACAGCTAGCTCAGTCCTAGG | To PCR-amplify the KO1-sgRNA construct |
| KO1-NotI-XhoI-sgRNA-R | TGCTGAAAAACTCGAAAAAAAAGCACCGACTCGGTGCCAC |  |
| KO1-ck-F | GACTGGTTAGAGTAACGCCC | To check KO1 mutant |
| KO1-ck-R | TCCATGATGTTGTCTCCGT |  |
| KO2-LA-F | CTCGAGTTTTTCAGCAAGATGGCTGCTCAACCGGACTTATC | To PCR-amplify the left region of B2M23_RS07870 |
| KO2-LA-R | AAACCACAAAACGTATTCCAGATCCTTATCGC |  |
| KO2-erm-F | TGGAATACGTTTTGTGGTTTATTTACAAATTCGGC | To PCR-amplify the *ermB* resistance cassette |
| KO2-erm-R | CCAGAAGCTCTGTTTGCAAGCAGCAGATTACGC |  |
| KO2-RA-F | CTTGCAAACAGAGCTTCTGGAGGACATGGG | To PCR-amplify the right region of B2M23_RS07870 |
| KO2-RA-R | AGGAGATCTTCTAGAAAGATAAGTTGGTGGGCATCTTGGT |  |
| KO2-sgOligo-F | TTGACAGCTAGCTCAGTCCTAGGTATAATACTAGTGAAGCTCCAGAGCCAGATTAGTTTTAGAGCTAGAAATAGCAAGTT | To prepare template for KO2-sgRNA |
| KO2- sgOligo-R | AAAAAAAGCACCGACTCGGTGCCACTTTTTCAAGTTGATAACGGACTAGCCTTATTTTAACTTGCTATTTCTAGCTCTAA |  |
| KO2-PstI-NotI-sgRNA-F | TCACGAGGCCGCCCCTTGACAGCTAGCTCAGTCCTAGG | To PCR-amplify the KO2-sgRNA construct |
| KO2-PstI-NotI-sgRNA-R | GAAGATCTGGCGGCCAAAAAAAGCACCGACTCGGTGCCAC |  |
| KO2-ck-F | CTGATCCAGCCGGTGAGTA | To check KO2 mutant |
| KO2-ck-R | TCTGAGCTGAGACATGCACT |  |
| KO3-LA-F | CTCGAGTTTTTCAGCAAGATAGGCCTTTGTTGTCGATGGt | To PCR-amplify the left region of B2M23_RS15135 |
| KO3-LA-R | AAACCACAAATGCCATCACACGTTCTCTCG |  |
| KO3-erm-F | TGTGATGGCATTTGTGGTTTATTTACAAATTCGGC | To PCR-amplify the *ermB* resistance cassette |
| KO3-erm-R | ACAGGCACCCTGTTTGCAAGCAGCAGATTACGC |  |
| KO3-RA-F | CTTGCAAACAGGGTGCCTGTGGATATGGtc | To PCR-amplify the right region of B2M23_RS15135 |
| KO3-RA-R | AGGAGATCTTCTAGAAAGATCGCCGAATTCCTGTTTCAGC |  |
| KO3-sgOligo-F | TTGACAGCTAGCTCAGTCCTAGGTATAATACTAGTGCACCCGGTCGGGCTCCTGGGTTTTAGAGCTAGAAATAGCAAGTT | To prepare template for KO3-sgRNA |
| KO3- sgOligo-R | AAAAAAAGCACCGACTCGGTGCCACTTTTTCAAGTTGATAACGGACTAGCCTTATTTTAACTTGCTATTTCTAGCTCTAA |  |
| KO3-NotI-XhoI-sgRNA-F | ATAGGGAGAGCGGCCTTGACAGCTAGCTCAGTCCTAGG | To PCR-amplify the KO3-sgRNA construct |
| KO3-NotI-XhoI-sgRNA-R | TGCTGAAAAACTCGAAAAAAAAGCACCGACTCGGTGCCAC |  |
| KO4-LA-F | CTCGAGTTTTTCAGCAAGATAGAGGACTGTCCCATCTGCCA | To PCR-amplify the left region of B2M23_RS16860 |
| KO4-LA-R | AAACCACAAATTCGGGCAGGAACCGTAA |  |
| KO4-erm-F | CCTGCCCGAATTTGTGGTTTATTTACAAATTCGGC | To PCR-amplify the *ermB* resistance cassette |
| KO4-erm-R | GGTGACTGGGTGTTTGCAAGCAGCAGATTACGC |  |
| KO4-RA-F | CTTGCAAACACCCAGTCACCACCAAGAGC | To PCR-amplify the right region of B2M23_RS16860 |
| KO4-RA-R | AGGAGATCTTCTAGAAAGATTACTCCAGGTATCGGCCACC |  |
| KO4-sgOligo-F | TTGACAGCTAGCTCAGTCCTAGGTATAATACTAGTTCTTGGTGGTGACTGGGTTCGTTTTAGAGCTAGAAATAGCAAGTT | To prepare template for KO4-sgRNA |
| KO4- sgOligo-R | AAAAAAAGCACCGACTCGGTGCCACTTTTTCAAGTTGATAACGGACTAGCCTTATTTTAACTTGCTATTTCTAGCTCTAA |  |
| KO4-NotI-XhoI-sgRNA-F | ATAGGGAGAGCGGCCTTGACAGCTAGCTCAGTCCTAGG | To PCR-amplify the KO4-sgRNA construct |
| KO4-NotI-XhoI-sgRNA-R | TGCTGAAAAACTCGAAAAAAAAGCACCGACTCGGTGCCAC |  |
| KO4-ck-F | TCATCAATTCCCTGGTCAGC | To check KO4 mutant |
| KO4-ck-R | GCCAGTTTGGTTCTCAGGAA |  |
| Bact1369-F | CGGTGAATACGTTCCCGG | To PCR-amplify bacterial 16S rRNA genes |
| Prok1492-R | TACGGCTACCTTGTTACGACTT |  |
| TM1389-P | 6FAM-CTTGTACACACCGCCCGTC |  |

Table S5. Chemicals used in this study.

| **Name** | **Source** | **Cat. No.** |
| --- | --- | --- |
| Acitretin | TargetMol | T1330 |
| Agomelatine | TargetMol | T1445 |
| Alfuzosin HCl | TargetMol | T0091 |
| Aliskiren hemifumarate | TargetMol | T1520 |
| Amisulpride | TargetMol | T0811 |
| Anisindione | Chem-Impex International | 10723 |
| Apixaban | TargetMol | T1736 |
| Avanafil | TargetMol | T2334 |
| Bosentan | TargetMol | T6264 |
| Bosutinib | TargetMol | T0152 |
| Bromopride | TargetMol | T1175 |
| Carvedilol | TargetMol | T0447 |
| Cisapride | TargetMol | T1291 |
| Codeine | Millipore-Sigma (Supelco) | C-006 |
| Colchicine | TargetMol | T0320 |
| Dextromethorphan | TargetMol | T0262L2 |
| Dihydrocodeine HCl | Millipore-Sigma (Supelco) | D-019 |
| Diltiazem HCl | TargetMol | T0112 |
| Donepezil | TargetMol | T7041 |
| Doxazosin mesylate | TargetMol | T0101 |
| Esomeprazole magnesium | TargetMol | T2686 |
| Etoposide | Sigma-Aldrich | E1383 |
| Fostamatinib | TargetMol | T6115 |
| Galantamine HBr | TargetMol | T0086 |
| Gefitinib | TargetMol | T1181 |
| Gliquidone | TargetMol | T0371 |
| Glyburide | TargetMol | T1634 |
| Griseofulvin | TargetMol | T1382 |
| Guaifenesin | TargetMol | T0739 |
| Hydrocodone | Millipore-Sigma (Supelco) | H-003 |
| Iloperidone | TargetMol | T1539 |
| Indomethacin | TargetMol | T0458 |
| Levomepromazine | MedChemExpress | HY-B1693 |
| Melatonin | TargetMol | T1659 |
| Mestranol | TargetMol | T1579 |
| Methicillin sodium | MedChemExpress | HY-B0974 |
| Methocarbamol | TargetMol | T1409 |
| Methoxsalen | TargetMol | T1548 |
| Metoclopramide HCl | TargetMol | T1069 |
| Midodrine HCl | Millipore-Sigma (Supelco) | M8277 |
| Moexipril HCl | TargetMol | T6595 |
| Moxifloxacin HCl | TargetMol | T0331 |
| Mycophenolic Acid | TargetMol | T1335 |
| Nabumetone | TargetMol | T1258 |
| Naproxen | TargetMol | T1582 |
| Omeprazole | TargetMol | T0757 |
| Oxycodone | Millipore-Sigma (Supelco) | O-002 |
| Papaverine HCl | Sigma-Aldrich | P3510 |
| Prazosin HCl | TargetMol | T1050 |
| Primaquine diphosphate | TargetMol | T0850 |
| Pyrilamine maleate | TargetMol | T1232 |
| Quinacrine dihydrochloride | TargetMol | T0942 |
| Quinidine | TargetMol | T7938 |
| Quinine | TargetMol | T0690 |
| Ranolazine | TargetMol | T6633 |
| Remoxipride HCl | Tocris | 0916 |
| Rescinnamine | Toronto Research Chemicals | R144720 |
| Simeprevir | TargetMol | T4686 |
| Sinensetin | TargetMol | T4S0227 |
| Sulpiride | TargetMol | T1201 |
| Tamsulosin HCl | TargetMol | T0210 |
| Tangeretin | TargetMol | T2842 |
| Terazosin HCl | TargetMol | T0197 |
| Tetrabenazine | TargetMol | T0719 |
| *cis*-Tramadol HCl | Millipore-Sigma (Supelco) | T-027 |
| Trimethobenzamide HCl | TargetMol | T5009 |
| Trimethoprim | TargetMol | T1153 |
| Vandetanib | TargetMol | T1656 |
| Venlafaxine | TargetMol | T22453 |
| Verapamil HCl | TargetMol | T1010 |
| Zafirlukast | TargetMol | T6736 |

Table S6. HPLC-UV protocols used in Figure 1c.

| **Compound** | **Wavelength (nm)** | **Gradient** | **Mobile phase A** | **Mobile phase B** | **Retention time (min)** |
| --- | --- | --- | --- | --- | --- |
| Agomelatine | 254 | 0-20 mins: 15-90% B,  20-30 mins: 90-90% B,  30-35 mins: 90-15% B,  35-45 mins: 15-15% B | 10 mM ammonium acetate in water | Acetonitrile | 14.6 |
| Alfuzosin | 248 | 0-20 mins: 15-90% B,  20-30 mins: 90-90% B,  30-35 mins: 90-15% B,  35-45 mins: 15-15% B | 10 mM ammonium acetate in water | Acetonitrile | 9.2 |
| Amisulpride | 227 | 0-20 mins: 15-90% B,  20-30 mins: 90-90% B,  30-35 mins: 90-15% B,  35-45 mins: 15-15% B | 10 mM ammonium acetate in water | Acetonitrile | 7.9 |
| Anisindione | 245 | 0-20 mins: 15-90% B,  20-30 mins: 90-90% B,  30-35 mins: 90-15% B,  35-45 mins: 15-15% B | 10 mM ammonium acetate in water | Acetonitrile | 10.4 |
| Apixaban | 280 | 0-20 mins: 15-90% B,  20-30 mins: 90-90% B,  30-35 mins: 90-15% B,  35-45 mins: 15-15% B | Water | Acetonitrile | 12.3 |
| Avanafil | 245 | 0-20 mins: 15-90% B,  20-30 mins: 90-90% B,  30-35 mins: 90-15% B,  35-45 mins: 15-15% B | 10 mM ammonium acetate in water | Acetonitrile | 14.6 |
| Bosutinib | 250 | 0-20 mins: 15-90% B,  20-30 mins: 90-90% B,  30-35 mins: 90-15% B,  35-45 mins: 15-15% B | 10 mM ammonium acetate in water | Acetonitrile | 14.0 |
| Carvedilol | 285 | 0-20 mins: 15-90% B,  20-30 mins: 90-90% B,  30-35 mins: 90-15% B,  35-45 mins: 15-15% B | 10 mM ammonium acetate in water | Acetonitrile | 14.6 |
| Codeine | 284 | 0-20 mins: 15-90% B,  20-30 mins: 90-90% B,  30-35 mins: 90-15% B,  35-45 mins: 15-15% B | 10 mM ammonium acetate in water | Acetonitrile | 7.5 |
| Colchicine | 330 | 0-20 mins: 15-90% B,  20-30 mins: 90-90% B,  30-35 mins: 90-15% B,  35-45 mins: 15-15% B | Water | Acetonitrile | 10.6 |
| Dihydrocodeine | 282 | 0-20 mins: 15-90% B,  20-30 mins: 90-90% B,  30-35 mins: 90-15% B,  35-45 mins: 15-15% B | 10 mM ammonium acetate in water | Acetonitrile | 5.7 |
| Diltiazem | 238 | 0-20 mins: 15-90% B,  20-30 mins: 90-90% B,  30-35 mins: 90-15% B,  35-45 mins: 15-15% B | 10 mM ammonium acetate in water | Acetonitrile | 15.2 |
| Donepezil | 268 | 0-20 mins: 15-90% B,  20-30 mins: 90-90% B,  30-35 mins: 90-15% B,  35-45 mins: 15-15% B | 10 mM ammonium acetate in water | Acetonitrile | 13.2 |
| Doxazosin | 244 | 0-20 mins: 15-90% B,  20-30 mins: 90-90% B,  30-35 mins: 90-15% B,  35-45 mins: 15-15% B | 10 mM ammonium acetate in water | Acetonitrile | 14.4 |
| Etoposide | 210 | 0-30 mins: 15-35% B,  30-36 mins: 95-95% B,  36-42 mins: 15-15% B | Water | Acetonitrile | 30.8 |
| Fostamatinib | 254 | 0-20 mins: 15-90% B,  20-30 mins: 90-90% B,  30-35 mins: 90-15% B,  35-45 mins: 15-15% B | 10 mM ammonium acetate in water | Acetonitrile | 10.5 |
| Galantamine | 290 | 0-20 mins: 15-90% B,  20-30 mins: 90-90% B,  30-35 mins: 90-15% B,  35-45 mins: 15-15% B | 10 mM ammonium acetate in water | 10 mM ammonium acetate in acetonitrile | 20.3 |
| Griseofulvin | 291 | 0-20 mins: 15-90% B,  20-30 mins: 90-90% B,  30-35 mins: 90-15% B,  35-45 mins: 15-15% B | Water | Acetonitrile | 15.4 |
| Hydrocodone | 284 | 0-20 mins: 15-90% B,  20-30 mins: 90-90% B,  30-35 mins: 90-15% B,  35-45 mins: 15-15% B | 10 mM ammonium acetate in water | Acetonitrile | 8.2 |
| Levomepromazine | 330 | 0-20 mins: 15-90% B,  20-30 mins: 90-90% B,  30-35 mins: 90-15% B,  35-45 mins: 15-15% B | 10 mM ammonium acetate in water | Acetonitrile | 16.5 |
| Methoxasalen | 249 | 0-20 mins: 15-90% B,  20-30 mins: 90-90% B,  30-35 mins: 90-15% B,  35-45 mins: 15-15% B | 10 mM ammonium acetate in water | Acetonitrile | 14.6 |
| Metoclopramide | 272 | 0-20 mins: 15-90% B,  20-30 mins: 90-90% B,  30-35 mins: 90-15% B,  35-45 mins: 15-15% B | 10 mM ammonium acetate in water | Acetonitrile | 8.6 |
| Moexipril | 280 | 0-20 mins: 15-90% B,  20-30 mins: 90-90% B,  30-35 mins: 90-15% B,  35-45 mins: 15-15% B | 0.1% formic acid in water | 0.1% formic acid in acetonitrile | 13.3 |
| Papaverine | 254 | 0-20 mins: 15-90% B,  20-30 mins: 90-90% B,  30-35 mins: 90-15% B,  35-45 mins: 15-15% B | 10 mM ammonium acetate in water | Acetonitrile | 14.4 |
| Prazosin | 260 | 0-20 mins: 15-90% B,  20-30 mins: 90-90% B,  30-35 mins: 90-15% B,  35-45 mins: 15-15% B | 10 mM ammonium acetate in water | Acetonitrile | 10.8 |
| Ranolazine | 272 | 0-20 mins: 15-90% B,  20-30 mins: 90-90% B,  30-35 mins: 90-15% B,  35-45 mins: 15-15% B | 10 mM ammonium acetate in water | Acetonitrile | 14.0 |
| Rescinnamine | 217 | 0-20 mins: 15-90% B,  20-30 mins: 90-90% B,  30-35 mins: 90-15% B,  35-45 mins: 15-15% B | 10 mM ammonium acetate in water | Acetonitrile | 19.7 |
| Simeprevir | 254 | 0-20 mins: 15-90% B,  20-30 mins: 90-90% B,  30-35 mins: 90-15% B,  35-45 mins: 15-15% B | 10 mM ammonium acetate in water | Acetonitrile | 22.8 |
| Sinesetin | 330 | 0-20 mins: 15-90% B,  20-30 mins: 90-90% B,  30-35 mins: 90-15% B,  35-45 mins: 15-15% B | 10 mM ammonium acetate in water | Acetonitrile | 15.0 |
| Tamsulosin | 248 | 0-20 mins: 15-90% B,  20-30 mins: 90-90% B,  30-35 mins: 90-15% B,  35-45 mins: 15-15% B | 10 mM ammonium acetate in water | Acetonitrile | 11.8 |
| Tangeretin | 330 | 0-20 mins: 15-90% B,  20-30 mins: 90-90% B,  30-35 mins: 90-15% B,  35-45 mins: 15-15% B | 10 mM ammonium acetate in water | Acetonitrile | 17.6 |
| Terazosin | 248 | 0-20 mins: 15-90% B,  20-30 mins: 90-90% B,  30-35 mins: 90-15% B,  35-45 mins: 15-15% B | 10 mM ammonium acetate in water | Acetonitrile | 9.2 |
| *cis*-Tramadol | 284 | 0-20 mins: 15-90% B,  20-30 mins: 90-90% B,  30-35 mins: 90-15% B,  35-45 mins: 15-15% B | 10 mM ammonium acetate in water | Acetonitrile | 9.4 |
| Trimethobenzamide | 284 | 0-20 mins: 15-90% B,  20-30 mins: 90-90% B,  30-35 mins: 90-15% B,  35-45 mins: 15-15% B | 10 mM ammonium acetate in water | Acetonitrile | 10.0 |
| Trimethoprim | 210 | 0-30 mins: 15-35% B,  30-36 mins: 95-95% B,  36-42 mins: 15-15% B | 10 mM ammonium acetate in water | Acetonitrile | 16.6 |
| Venlafaxine | 223 | 0-20 mins: 15-90% B,  20-30 mins: 90-90% B,  30-35 mins: 90-15% B,  35-45 mins: 15-15% B | 10 mM ammonium acetate in water | Acetonitrile | 11.2 |
| Verapamil | 278 | 0-20 mins: 15-90% B,  20-30 mins: 90-90% B,  30-35 mins: 90-15% B,  35-45 mins: 15-15% B | 10 mM ammonium acetate in water | Acetonitrile | 15.7 |
